# Foam fractionation Tags (F-Tags) enabling surfactant-free, activity-preserving recovery of enzymes

**DOI:** 10.1007/s00253-023-12837-1

**Published:** 2024-01-17

**Authors:** Thomas Krause, Behnam Keshavarzi, Sascha Heitkam, Marion B. Ansorge-Schumacher

**Affiliations:** 1 Department of Molecular Biotechnology, TU Dresden, 01062 Dresden, Germany; 2 Institute of Process Engineering and Environmental Technology, TU Dresden, 01062 Dresden, Germany

**Keywords:** Downstream processing, Foam fractionation, Enzyme purification, Activity preservation

## Abstract

**Abstract:**

Enzymes have become important tools in many industries. However, the full exploitation of their potential is currently limited by a lack of efficient and cost-effective methods for enzyme purification from microbial production. One technology that could solve this problem is foam fractionation. In this study, we show that diverse natural foam-stabilizing proteins fused as F-Tags to β-lactamase, penicillin G acylase, and formate dehydrogenase, respectively, are able to mediate foaming and recovery of the enzymes by foam fractionation. The catalytic activity of all three candidates is largely preserved. Under appropriate fractionation conditions, especially when a wash buffer is used, some F-Tags also allow nearly complete separation of the target enzyme from a contaminating protein. We found that a larger distance between the F-Tag and the target enzyme has a positive effect on the maintenance of catalytic activity. However, we did not identify any particular sequence motifs or physical parameters that influenced performance as an F-tag. The best results were obtained with a short helical F-Tag, which was originally intended to serve only as a linker sequence. The findings of the study suggest that the development of molecular tags that enable the establishment of surfactant-free foam fractionation for enzyme workup is a promising method.

**Key points:**

*• Foam-stabilizing proteins mediate activity-preserving foam fractionation of enzymes*

*• Performance as an F-Tag is not restricted to particular structural motifs*

*• Separation from untagged protein benefits from low foam stability and foam washings*

**Graphical Abstract:**

**Figure:**
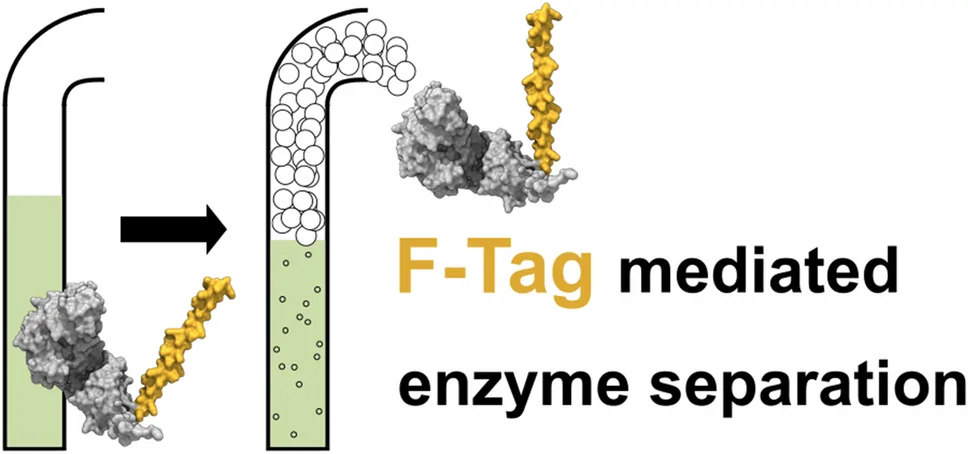

**Supplementary Information:**

The online version contains supplementary material available at 10.1007/s00253-023-12837-1.

## Introduction

Today, enzymes are important tools in numerous industrial sectors. Their application potential as efficient and specific catalysts, however, is far from being exploited. Especially for the synthesis of chiral compounds, special diagnostics or therapeutics, a significant bottleneck results from the availability of pure but not too expensive enzyme preparations. This correlates with the current lack of suitable methods for gentle and at the same time cost-efficient workup from microbial cultures and crude extracts.

From a technological point of view, foam fractionation as first described by Lemlich (1968) is an attractive approach to the development of enzyme downstream processing from microbial production. In this technique, target molecules adsorb to the gas-liquid interfaces of rising gas bubbles injected into their aqueous solution, and stabilize them by forming cohesive films. The bubbles rise as a foam inside a column. Physical processes (e.g., drainage, coalescence, and disproportionation) in the rising foam reduce the amount of entrained liquid and the foam volume, and enable enrichment of the adsorbed molecules. The target molecules are regained by foam collection and reliquefication, e.g., by application of vacuum, ultrasound, or stirring. For target molecules not able to stabilize foam, addition of surface-active foaming agents (e.g., surfactants) can achieve foam stability. Since individual structural features govern the tendency of molecules to adsorb at gas-liquid interfaces, the method in principle can achieve both enrichment and separation of specific target molecules from dilute solutions. Addition of a washing solution from above can improve the purity by removing molecules that are hydrodynamically entrapped in the foam (Keshavarzi et al. 2022a). Overall, mild operation conditions, minor energy and investment costs, lack of organic solvents, short processing times, and simple construction of devices distinguish the method and predestine it for integration into bioprocessing. In fact, foam fractionation has been studied extensively with regard to the purification of surface-active products from aerobic fermentation processes and has been introduced to a couple of processes as recently reviewed (Oraby et al. 2022).

It was shown years ago that proteins can be purified by foam fractionation (Linke and Berger 2011). Nevertheless, the number of enzymes for which this technique has been successfully demonstrated is small (about 20). To our knowledge, no technical application exists. From the common understanding of enzyme function, the interaction of proteins with gas-liquid interfaces, and the demands on enzyme preparation for technical use, we assume that two main factors are responsible for that failure: (1) direct adsorption of the target enzymes to the gas-liquid interface resulting in denaturation and aggregation (Cumper and Alexander 1950), and consequently severe loss of catalytic activity; (2) stabilization of gas-liquid interfaces (foam) with surfactants, which can act as effective inhibitors of enzyme function (Walker and McCallion 1980; Wang et al. 2009) and, probably even more importantly, require cost-intensive measures for subsequent separation from the final product (Wang et al. 2009). Accordingly, driving foam fractionation towards an efficient method for enzyme workup requires novel concepts for both the adsorption of enzymes to the interface and the stabilization of foam.

In a previous study, we demonstrated that fusion of β-lactamase (Bla) or penicillin G acylase (PGA) with Ranaspumin-2 (Rsn-2) enabled both enrichment of the enzymes in foam and foam stabilization without the need for special foaming agents (Krause et al. 2022). At the same time, the catalytic activity of both enzymes was widely retained during the foaming process. For Bla at least, this was clearly a credit to Rsn-2. Thus, tagging enzymes with an appropriate protein might be the clue to their surfactant-free and activity-preserving foam fractionation. The tag might even promote the selectivity of surficial protein enrichment considerably and act as a tool for enzyme foam fractionation independent of individual molecular features of the target enzyme itself.

In this study, we set the goal to identify important molecular properties of proteins that, as tags for foam fractionation of enzymes (F-Tags), enable stabilization of foam and recovery of enzymes as efficiently as possible while preserving catalytic activity. The tags should also allow separation of the target enzymes from other proteins. Towards this end, we selected a range of promising proteins, fused them to Bla individually, and investigated their effects on the enzyme and their performance after foaming in a simple column. As most of the fusion constructs heavily aggregated during microbial production, we introduced the Spy system (Zakeri et al. 2012) to link the different tags to the enzyme; in some cases, also the introduction of an additional linker between F-Tag and Bla was beneficial. We interpreted our observations with regard to possible connections between the tag effects and their molecular characteristics using structural models. Based on the experimental results, we selected three F-Tag candidates and investigated their effects on the separation of the fusion proteins from native eGFP. Finally, we demonstrated transferability of the performance of these three F-Tags with PGA and a formate dehydrogenase, both industrially relevant enzymes.

## Materials and methods

### Gene cloning and expression

 Plasmid propagation and cloning were performed using *E. coli* TOP10 (Thermo Scientific™, USA). Competent *E. coli* cells were transformed with standard electroporation methods. Genes coding for Bla, RjFDH, NrdJ-S, Rsn-2 (Addgene plasmid # 61686), SpyTag, and SpyCatcher (Addgene plasmid # 35044) were amplified from available plasmids (Mackenzie et al. 2009; Zakeri et al. 2012; Loderer et al. 2017; Boldt and Ansorge-Schumacher 2020); genes coding for SlyD, PGA, and BslA were amplified from genomic DNA of *E. coli* K12, *Kluyvera cryocrescens* (DSM No.: 2660), or *Bacillus subtilis W168*, respectively. Synthetic genes coding for ChpE, HsbA, R1, and R1-RdlA were obtained from Twist Bioscience (USA). All genes were cloned into commercial pET28 vectors via Gibson assembly (Gibson et al. 2009).

**Fig. 1:**
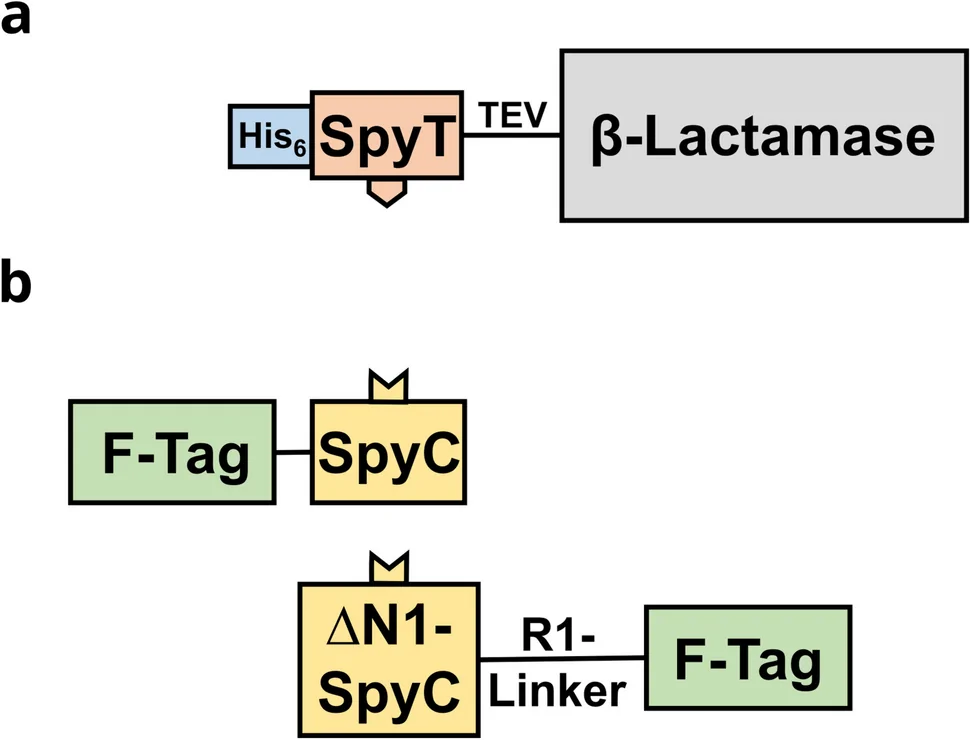
Construction setup for fusion of β-lactamase (Bla) to F-Tags mediated by the Spy system. **a** Histidine (His)_6_-tagged SpyTag (SpyT) N-terminally fused to Bla via a linker containing a protease (TEV) cleavage site. **b** SpyCatcher (SpyC) N-terminally or C-terminally fused to a F-Tag, occasionally via R1-linker

Constructs coding for Bla, PGA, or RjFDH contained N-terminally the coding sequence for SpyTag interspaced by the linker GSGGSG and a TEV protease cleaving site. The N-terminus also carried a *his*_6_ extension (Fig. 1a). Constructs coding for BslA, NrdJ-S, Rsn-2, or SlyD contained C-terminally the coding sequence for SpyCatcher (SpyC). For ChpE and HsbA, the coding sequence for ∆N1SpyC was employed instead of the original SpyC and was fused N-terminally. Constructs using the R1-Linker to interspace F-Tag candidates and SpyC used the coding sequence for ∆N1SpyC N-terminally (Fig. 1b).

For protein production, *E. coli* NiCo21 (DE3) competent cells (New England Biolabs GmbH, Germany) were transformed with the individual plasmids. All proteins were produced in TB-medium (24 g L^−1^ yeast extract, 12 g L^−1^ tryptone, 2.5 g L^−1^ glycerol, 0.89 mmol L^−1^ potassium phosphate, pH 7.3) containing kanamycin (50 μg mL^−1^) for selection. A fresh overnight culture was used to inoculate 1 L medium to an OD_600_ of 0.1. Cells grew at 37 °C under constant shaking until an OD_600_ of 0.5 was reached. After cooling this culture on ice for 10 min, gene expression was induced by addition of IPTG to a final concentration of 1 mmol L^−1^, and the culture was incubated for an additional 16 h at 16 °C with constant shaking.

### Protein isolation and purification

 Cells were harvested by centrifugation, suspended in 35 mL buffer solution (100 mmol L^−1^ KPi and 20 mmol L^−1^ imidazole, 0.5 mol L^−1^ NaCl, pH 7.5), and subjected to a French pressure cell at 1400 bar (internal cell pressure) after addition of 2.5 mg DNase I. Crude protein extract was obtained from centrifugation. Fusion proteins were created by mixing crude extract of enzymes tagged with SpyT and F-Tag candidates tagged with SpyC at a volumetric ratio of 2:1 at 4 °C for 15 min. The fusion proteins were then purified via immobilized metal ion affinity chromatography (IMAC) and size exclusion chromatography. The crude extracts were loaded on a HisTrap™ HP column (Cytiva, USA), washed with buffer solution as detailed above, and eluted with another buffer solution (100 mmol L^−1^ KPi and 500 mmol L^−1^ imidazole, 0.5 mol L^−1^ NaCl, pH 7.5). Elution fractions from the IMAC were then loaded on a HiLoad™ 16/600 Superdex™ 200 pg (Cytiva, USA) size exclusion column and run with SEC buffer (100 mmol L^−1^ KPi, pH 7.5). Purity was verified via SDS-PAGE. An exemplary result of a SEC purification is shown in Fig. S10.

### Determination of protein concentration

 The total protein concentration was determined using the Pierce™ BCA Protein Assay Kit (Thermo Scientific™, USA) according to the manufacturer’s instructions for the application in Microplates. The concentration of eGFP (*c*_*eGFP*_) was determined by measuring the adsorption of standard solutions (concentration range 0–0.2 mg mL^−1^) and sample solutions at 488 nm and applying Lambert–Beer’s law. Enzyme concentrations of Bla, PGA, or RjFDH (*c*_*Enzyme*_) in mixed solutions with eGFP were calculated as the difference of total protein and *c*_*eGFP*_.

### Determination of enzyme activity

 Bla specific activity (*A*_*Bla*_) was determined photometrically as described elsewhere (Bebrone et al. 2001). CENTA (Sigma-Aldrich/Merck, Germany) was used as substrate and the change in absorption was measured at 405 nm (*ε* 6400 L mol^−1^ cm^−1^) over 1 min. The activity test consisted of 920 μL KPi buffer (100 mmol L^−1^, pH 7.5), 70 μL CENTA (5 mmol L^−1^), and 10 μL sample solution.

The influence of the different tags and their varying molecular masses on the activity of Bla is described by the initial catalytic activity $\delta {\overline{A}}_{initial}=\frac{k_{tagged\ Bla}}{k_{untagged\ Bla}}$ with *k*_*Enzyme*_ = (*A*_*Enzyme*_ · *M*_*Enzyme*_).

PGA specific activity (*A*_*PGA*_) was determined photometrically with NIPAB (abcr GmbH, Germany) as substrate measuring the change in absorption at 405 nm (*ε* 9090 L mol^−1^ cm^−1^) over 1 min. The activity test consisted of 800 μL KPi buffer (100 mmol L^−1^, pH 7.5), 100 μL NIPAB (5 mmol L^−1^), and 100 μL sample solution.

RjFDH specific activity (*A*_*RjFDH*_) was determined photometrically via NAD^+^ reduction (Sigma-Aldrich/Merck, Germany) measuring the change in absorption at 340 nm (*ε* 6300 L mol^−1^ cm^−1^) over 1 min. The activity test consisted of 775 μL KPi buffer (100 mmol L^−1^, pH 7.5), 100 μL potassium formate (1 mol L^−1^), 25 μL NAD^+^ (100 mmol L^−1^), and 100 μL sample solution.

### Prediction of protein structures

 Structure prediction was performed using AlphaFold2 with the ColabFold interface (v1.5.1, (Mirdita et al. 2022)) and the default settings (num_relax = 0, template_mode = none, msa_mode = mmseqs2_uniref_env, pair_mode = unpaired_paired). SpyTag/SpyCatcher constructs were entered as monomers. Visual representations were generated using UCSF ChimeraX 1.5 (Pettersen et al. 2021).

### Determination of dynamic surface tension

 The maximum bubble pressure device BP100 (KRÜSS GmbH, Germany) was used to determine the dynamic surface tension *σ* in the time range of 10 ms–200 s. For the tests, a standard tube of 0.2-mm inner diameter with an immersion depth of 5 mm was used. The measurement for each point was conducted three times and an average value was reported.

### Determination of foam height

 Foam height was determined using the dynamic foam analyzer DFA100 (KRÜSS GmbH, Germany). The device had a cylindrical glass cell with 4-cm inner diameter and 24-cm height. A porous plate (provided from ROBU Glasfilter-Geraete GmbH, Germany) with 20-mm diameter and a thickness of 2.5 mm, mounted at the cell bottom, was used to disperse the air into the solution. The porous plate had pore sizes in the range of 160–250 μm. For the tests, 50 mL protein solution with varying concentrations was poured into the cell. Then, air was pumped through the solution with an air flow rate of 200 mL min^−1^ min until 100 mL of air was pumped. The maximum foam height was recorded in the cell. In the case of perfect foamability, the foam height reached 9 cm.

### Foaming and foam fractionation

 Foam stabilizing properties of proteins were determined in a glass column as illustrated in Fig. 3a, mounted on a Plexiglas base holding a porous plate (pore size 160-250 μm). The column had an internal diameter of 1.2 cm and a height of 15 cm. The air was dispersed into the column through a sintered glass porous plate (ROBU Glasfilter-Geraete GmbH, Germany) with a diameter of 20 mm and a thickness of 2.5 mm to produce rising bubbles. The airflow to the column was adjusted to 20 mL min^−1^ using an FMA5510A Gas Mass Controller (Omega Engineering, USA). For each experiment, 10 mL protein solution diluted in 100 mmol L^−1^ KPi buffer (pH 7.5) were used, filling the cell up to 8.6 cm. Samples were taken from the bulk solution before foaming (initial solution) and after the experiment (retentate) and from the liquefied foam overflow. The experiment ended when the foam overflow stopped. All samples were analyzed for protein concentration and enzyme activity.

For foam fractionation offering the possibility to add wash buffer, a glass column as illustrated in Fig. 3b was mounted on the same Plexiglas part as described above. The glass column had an internal diameter of 2.2 cm and a height of 16 cm. The overflow from the column was directed into two drains: the foam outlet, and the drain outlet that was used to drain the washing liquid. We used the same sintered glass porous plate as before for bubble generation. The airflow to the column was adjusted to 100 mL min^−1^ using a FMA5510A Gas Mass Controller (Omega Engineering, USA). A Fusion 4000 double syringe pump (KR Analytical Limited, UK) added the wash buffer (100 mmol L^−1^ KPi buffer (pH = 7.5)) with a flow rate of 0.5 mL min^−1^ at the top of the foam through a small opening (2-mm diameter) where the injection tube was connected to the glass column. For each experiment, 42 mL protein solution diluted in 100 mmol L^−1^ KPi buffer (pH = 7.5) was used, filling the cell up to 11.1 cm. Samples were taken from the bulk solution before foaming (initial solution) and after the experiment (retentate), from the drain outlet, and from the liquefied foam overflow. The experiment ended when the foam overflow stopped. All samples were analyzed for protein concentration (*c*) and enzyme activity (*A*).

The influence of the foaming process on enzyme activity is described by relative residual activity $\delta A=\frac{A_{overflow}}{A_{initial}}$. The protein enrichment $E=\frac{c_{overflow}}{c_{initial}}$ describes the increase in *c* after foaming. Multiplying with the corresponding liquid volume *V* yields the total mass recovery $R=\frac{V_{overflow}\,\cdot\,{c}_{overflow}}{V_{initial}\,\cdot\,{c}_{initial}}$. However, not all of the initial protein was found in the overflow or in the retentate. The protein loss equals *δm* = *V*_*initial*_*c*_*initial*_ − *V*_*overflow*_*c*_*overflow*_ − *V*_*retentate*_*c*_*retentate*_. In the case of protein mixtures A + B, the purity or grade of the target protein A is defined by $P=\frac{c_A}{\left({c}_A+{c}_B\right)\ }$.

## Results

### F-Tag candidates and fusion constructs

 On the hypothesis that the promising performance of Rsn-2 as a foam fractionation tag (Krause et al. 2022) was related to its natural function as a foam-stabilizing agent, we performed a literature research to identify proteins from prokaryotic and eukaryotic sources with known or assumed surface activity as potential F-Tag candidates. In view of their application for purification of recombinant enzymes derived from microbial hosts, we set a maximum size of 30 kDa and excluded proteins with post-translational modifications. Many of the identified proteins could not be produced in *E. coli* in soluble form, but we were able to obtain seven candidates for further study (Table 1). This included Rsn-2 from our previous work (Krause et al. 2022) as a positive control.

**Table 1:** Selection of F-Tag candidates referred to their natural sources and functions as described in literature or derived from own observations

F-Tag candidate	Full name	Size (kDa)	Source	Natural function	Reference
BslA	Biofilm surface layer protein A	15.1	*Bacillus subtilis* W168	Biofilm formation	Hobley et al. (2013)
ChpE	Chaplin E	7.8	*Streptomyces coelicolor*	Aerial hyphae formation	Claessen et al. (2003)
HsbA	Hydrophobic surface binding protein A	18.3	*Aspergillus oryzae*	Unknown	Ohtaki et al. (2006)
NrdJ (C-domain)	Vitamin B12-dependent ribonucleotide reductase	16.2	*Stackebrandtia nassauensis*	Ribonucleotide reductase	Loderer et al. (2017)
RdlA	Rodlin A	13.1	*Streptomyces coelicolor*	Aerial hyphae formation	Claessen et al. (2002)
Rsn-2	Ranaspumin-2	11.3	*Engystomops pustulosus*	Foam nest formation	Fleming et al. (2009)
SlyD	Sensitive to lyses D protein	20.9	*Escherichia coli*	Chaperone and peptidyl-prolyl cis-trans isomerase activity	Scholz et al. (2006) Found in a foam sample of cell extract.

Unlike previously with Rsn-2, most constructs obtained from a direct fusion of the genes encoding Bla and F-Tag candidate formed only insoluble aggregates. The sole exception was BslA. We solved this problem by the introduction of the Spy system, i.e., we produced Bla fused with SpyTag (SpyT) and F-Tags fused with SpyCatcher (SpyC) first separately and then covalently linked the two proteins by the spontaneous formation of an isopeptide bond between SpyT and SpyC upon mixing both components. The full constructs were appropriately soluble. Introduction of the Spy system enlarged the constructs by 17 kDa or 14 kDa, respectively, depending on the SpyC-variant included. For optimal comparability, we used the Spy system on all tags, including Rsn-2. SpyT was N-terminally fused to Bla, and a short linker sequence (GSGGSG) and a cleavage site for TEV protease were introduced between the two (Fig. 1a). Additionally, the construct was N-terminally equipped with a His_6_-Tag to enable purification with a conventional immobilized metal affinity chromatography (IMAC). SpyC (14.3 kDa) or its ∆N1-variant (11.3 kDa) was either C-terminally (BslA, NrdJ, Rsn-2, SlyD) or N-terminally (ChpE, HsbA, RdlA) fused to the F-Tag candidates depending on the solubility of the resulting proteins (Fig. 1b). Recombinant expression of *rdlA* required introduction of an additional linker between SpyC and RdlA to enhance gene expression and protein solubility. Four linker sequences described in literature (Chen et al. 2013) were tested for this purpose (data not shown) and the long rigid linker R1 with the sequence A(EAAAK)_4_ALEA(EAAAK)_4_A and a molecular size of 4.3 kDa was finally included.

**Fig. 2:**
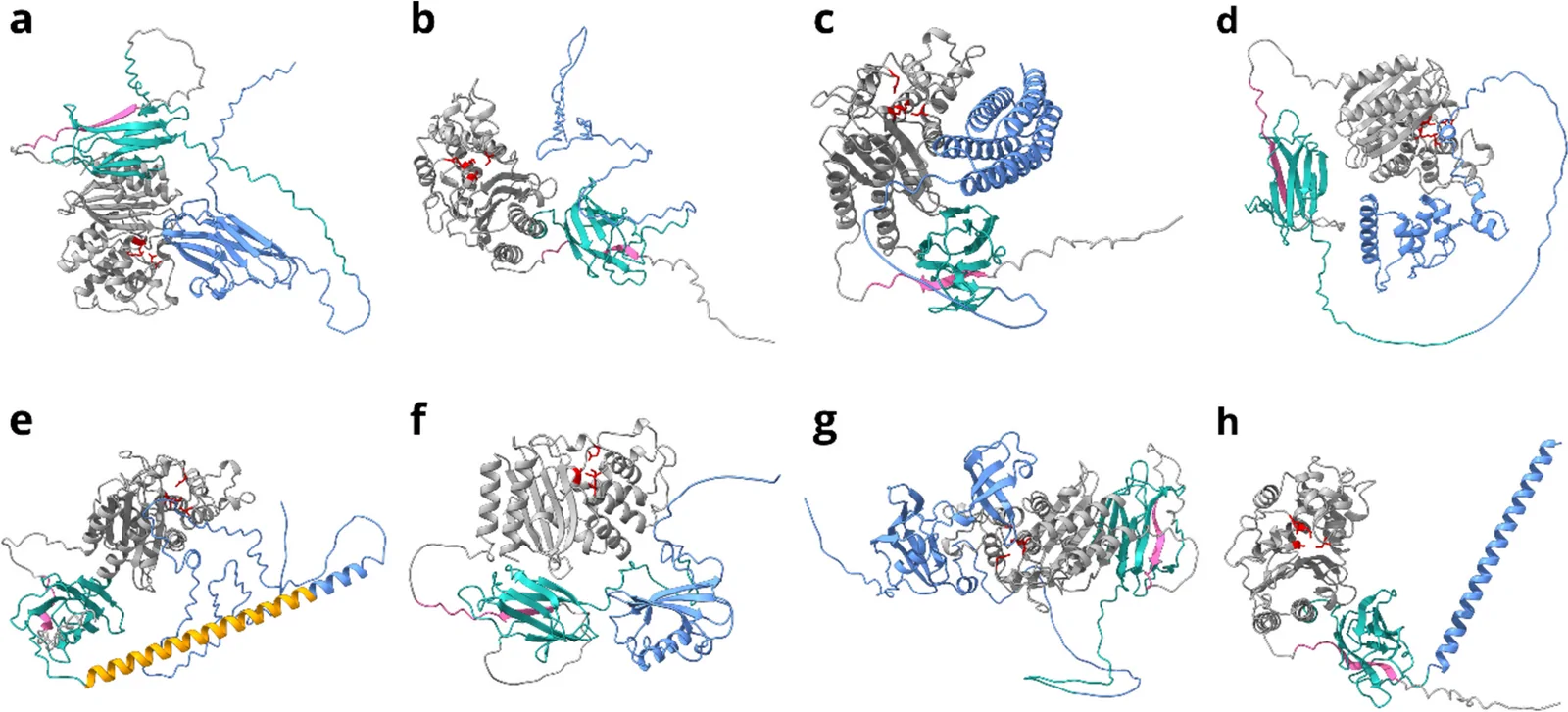
Structural models of F-Tag-Bla fusion constructs. green: SpyCatcher-domain; pink: SpyTag-domain; gray: Bla with catalytic residues (Ser70, Lys73, Ser130; Glu166 (Minasov et al. 2002)) in red; orange: R1-linker; blue: F-Tag: **a** BslA, **b** ChpE, **c** HsbA, **d** NrdJ-S, **e** R1-RdlA, **f** Rsn-2, **g** SlyD, **h** R1

We visualized the fusion constructs using AlphaFold2 (Fig. 2, Fig. S1) under separate consideration of the structures of the entities involved in the construct and under the assumption that the covalent bond between SpyT and SpyC dictated the orientation between Bla and F-Tag. All constructs showed an average predicted local-distance difference test value (pLDDT) > 70. The Predicted Aligned Error (PAE) was low for the individual domains (Bla, SpyT/SpyC, F-Tag), but considerably higher where the interactions between the domains were concerned. pLDDT and PAE plots are given in Fig. S2 and Fig. S3, respectively.

### Tag influences on enzyme activity and foaming performance

**Fig. 3:**
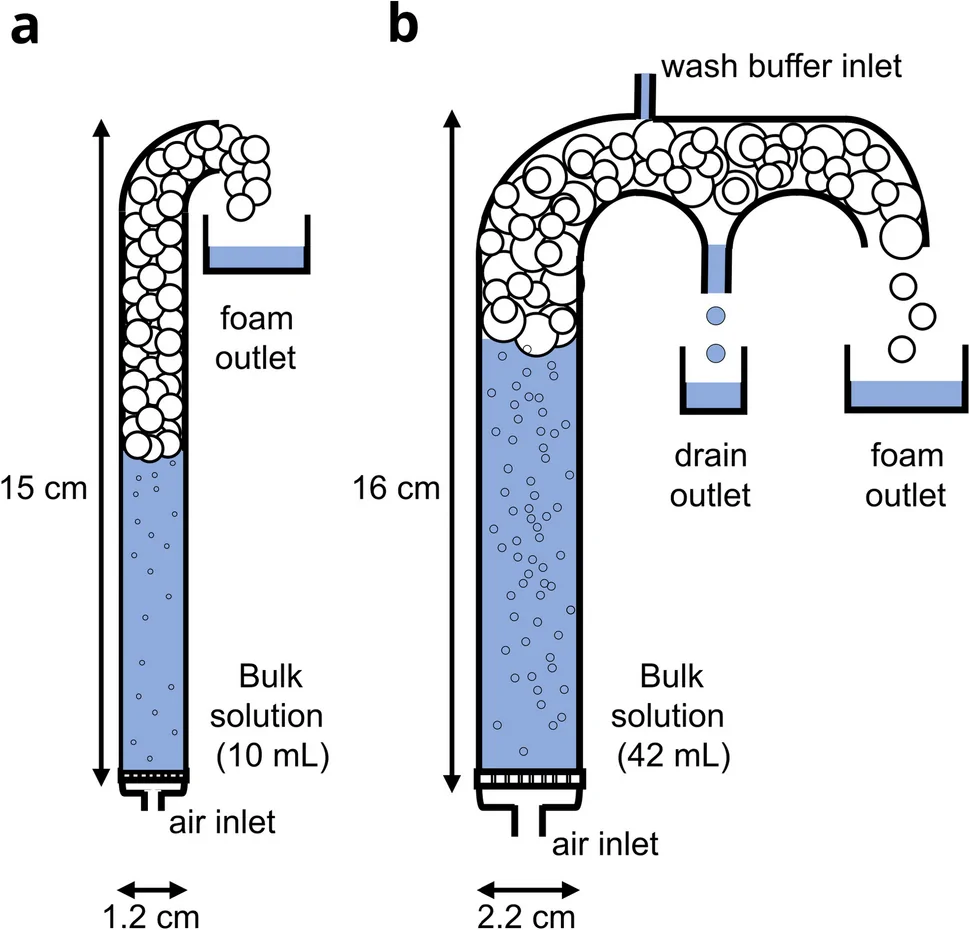
Column setup for foaming and foam fractionation experiments. **a** Simple column with air inlet and foam outlet. **b** Extended column with additional wash buffer inlet and drain outlet

 All fusion constructs were enzymatically active and were able to build and stabilize foam in a simple foam fractionation column (Fig. 3a). However the catalytic activities and protein recoveries varied considerably with the F-Tag involved (Table 2)

**Table 2:** Catalytic activity of Bla fusion constructs before $\delta {\overline{A}}_{initial}$ and after foaming  (δ*A*), protein loss  (δ*m*), enrichment (*E*), and protein recovery (*R*) after foaming in a standard column

Fusion protein	*M* (kDa)	$\delta {\overline{A}}_{initial}$ (%)	*δA* (%)	*δm* (%)	*E*	*R* (%)
Bla_untagged_	33.7	100	-	-	-	-
Bla-Rsn-2_original_ (directly fused; Krause et al. 2022)	43.5	26 ± 9	54 ± 10	24 ± 5	1.45 ± 0.07	17 ± 0
Initial constructs						
Bla-BslA	63.0	66 ± 6	**51 ± 7**	*62 ± 0*	0.86 ± 0.06	*15 ± 1*
Bla-ChpE	52.8	89 ± 1	**92 ± 2**	*42 ± 4*	1.62 ± 0.12	*16 ± 1*
Bla-HsbA	62.8	76 ± 2	*39 ± 2*	**29 ± 3**	2.95 ± 0.48	**52 ± 0**
Bla-NrdJ-S	64.1	55 ± 4	**59 ± 2**	**33 ± 1**	2.86 ± 0.02	*32 ± 0*
Bla-R1-RdlA	62.4	53 ± 1	**69 ± 5**	**23 ± 1**	3.13 ± 0.13	*37 ± 0*
Bla-Rsn-2	59.2	95 ± 3	*34 ± 3*	**6 ± 1**	3.66 ± 0.42	*22 ± 3*
Bla-SlyD	68.8	66 ± 5	**69 ± 4**	**16 ± 2**	2.17 ± 0.13	**64 ± 2**
Constructs extended with R1						
Bla-R1-HsbA	67.1	72 ± 4	**99 ± 5**	**24 ± 0**	1.86 ± 0.00	**52 ± 1**
Bla-R1-NrdJ-S	65.6	63 ± 2	**81 ± 8**	**35 ± 3**	1.45 ± 0.06	*34 ± 6*
Bla-R1-Rsn-2	60.5	100 ± 3	**64 ± 4**	**17 ± 10**	1.73 ± 0.27	**61 ± 11**
Bla-R1-SlyD	70.3	82 ± 3	**87 ± 5**	*40 ± 0*	1.12 ± 0.07	*21 ± 7*
Bla-R1	49.3	82 ± 6	**73 ± 4**	**20 ± 1**	1.95 ± 0.13	**64 ± 5**

Values worse than 40% are presented in italics and values better than 40% are presented in bold. Foaming was performed in the simple setup until no foam overflowed. Standard deviations refer to two separate experiments

In terms of initial catalytic activity $\delta {\overline{A}}_{initial}$, i.e., the activity of the fusion proteins relative to the untagged enzyme, the constructs performed best in the order (Bla-Rsn-2 ~ Bla-ChpE) > Bla-HsbA > (Bla-BslA ~ Bla-SlyD) > (Bla-NrdJ-S ~ Bla-R1-RdlA) (Table 2, upper part). With $\delta {\overline{A}}_{initial}$ around 90%, the first two, Bla-Rsn-2 and Bla-ChpE, were clearly outstanding, but even the activities of the worst constructs were still about 50%. Notably, all fusion proteins obtained with the help of the Spy system were considerably more active than Bla-Rsn-2_original_, which had a $\delta {\overline{A}}_{initial}$ of only 26% (Krause et al. 2022). In direct comparison, the new constructs of Bla-Rsn-2 showed a 3.5-fold increase in $\delta {\overline{A}}_{initial}$.

Considering residual activity after foaming, *δA*, i.e., enzymatic activity of protein dissolved in the liquefied foam relative to the initial activity in the reservoir solution, the fusion proteins performed in the order Bla-ChpE > (Bla-R1-RdlA ~ Bla-SlyD) > Bla-NrdJ-S > Bla-BslA > (Bla-HsbA ~ Bla-Rsn-2). Again, the activity of Bla-ChpE, as referred to the extracted and soluble protein, was about 90% and thus clearly outstanding. On the other hand, Bla-Rsn-2 showed the worst *δA* in this experiment, closely followed by Bla-HsbA, which ranked third with regard to $\delta {\overline{A}}_{initial}$. The difference between the best and worst constructs was as large as 57%.

Unfortunately, the best construct regarding both $\delta {\overline{A}}_{initial}$ and *δA*, Bla-ChpE, heavily precipitated during foaming. This accounted for a loss of soluble protein *δm* of nearly 42%, which prematurely disqualified this construct as a means for effective downstream processing. The same was true for Bla-BslA, of which even *δm* = 62% were lost during foaming. The other constructs showed no visible precipitation, even if *δm* was up to 40%.

On the other hand, the linker R1 seemed to have a positive influence on solubility of the fusion constructs and *δA*. *δA* of Bla-R1-RdlA after foaming was nearly 70% and thus second to best of all constructs, while *δm* was about 20%. The positive effect on *δA* during foaming was confirmed when we introduced R1 as a linker to Bla-HsbA, Bla-NrdJ-S, Bla-Rsn-2, and Bla-SlyD. *δA* of all constructs increased significantly by 59.8%, 21.7%, 30%, and 17.9%, respectively (Table 2, lower part), compared to the variant without linker. The influence on *δm*, however, was indifferent as it partly increased or decreased. For Bla-SlyD, the introduction of R1 increased *δm* considerably to almost 40%. Regarding $\delta {\overline{A}}_{initial}$, the presence of R1 had a distinctly positive effect on Bla-SlyD, but on Bla-Rsn-2 and Bla-NrdJ-S the effect was only slight, and the activities of the other fusion constructs hardly changed. Interestingly, fusion of only R1 to Bla also conveyed the ability to build and stabilize foam, sparsely affected $\delta {\overline{A}}_{initial}$, and achieved a good *δA* after foaming combined with a comparably low *δm*.

All constructs except Bla-BslA achieved enrichment during foaming, i.e., *E* > 1. The original constructs performed in the order Bla-Rsn-2 > (Bla-R1-RdlA ~ Bla-HsbA ~ Bla-NrdJ-S) > Bla-SlyD > Bla-ChpE. Despite the very high *E* with Bla-R1-RdlA, however, introducing R1 to the other constructs had a clearly negative effect. Nevertheless, there still was considerable enrichment of Bla fused only with R1.

**Fig. 4:**
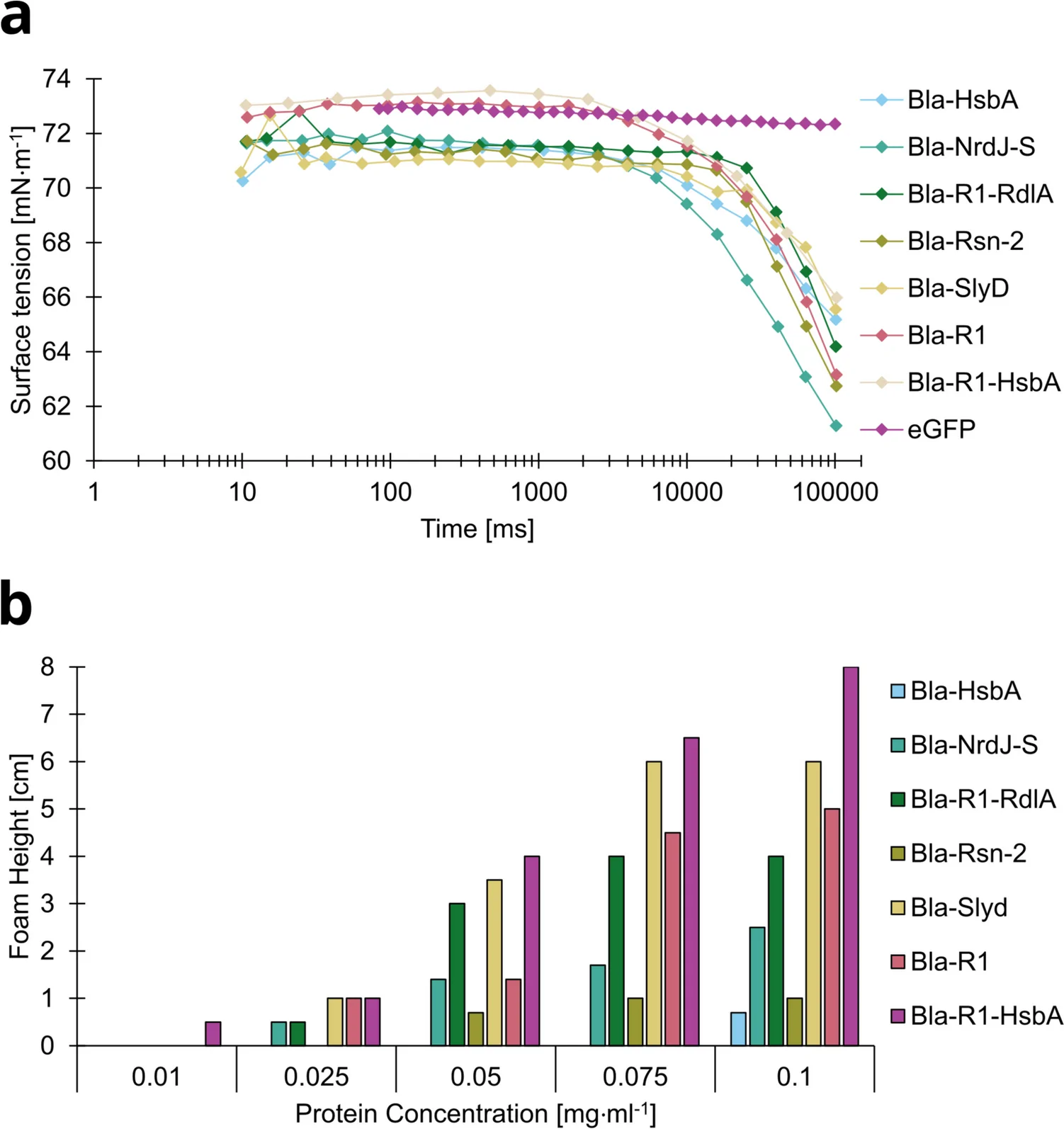
Surface activity of and foam stabilization with Bla-F-Tag constructs. **a** Dynamic surface tension of solutions containing 0.1 mg mL^−1^. **b** Foam height at different protein concentrations

Enrichment did not correlate with the protein recovery *R*, i.e., the mass of soluble protein in the liquefied foam related to the mass in the initial (bulk) solution. *R* followed the order Bla-SlyD > Bla-HsbA > (Bla-R1-RdlA ~ Bla-NrdJ-S) > Bla-Rsn-2 > (Bla-ChpE ~ Bla-BslA). We also found no correlation between the enrichment or recovery of the fusion constructs and their surface activity. As demonstrated in Fig. 4a, all tested constructs had comparable effects on surface tension. Particularly within the first seconds, which represent a fast adsorption in foam fractionation, differences were not significant. Slight differences occurred with regard to the time after which the surface tension started to decrease. However, the different fusion constructs distinctly supported different foam heights (Fig. 4b). The foam height increased with increasing concentration of the fusion proteins in the reservoir solution, but mostly reached a plateau at 0.075 mg mL^−1^. Only with Bla-HsbA, formation of a stable foam required a minimum concentration of 0.1 mg mL^−1^. In contrast, Bla-R1-HsbA required only 0.01 mg mL^−1^ to build a foam and overall gave the highest foam stability of nearly 90% residual foam volume. Generally, introduction of R1 increased the residual foam height (Fig. 4b). Bla-R1 without an additional tag performed very well. Again a correlation between foamability and *E* or *R*, respectively, did not occur.

Since none of the Bla-F-Tag constructs showed a clear outstanding performance with all the investigated parameters, we decided that residual activity *δA*, protein loss *δm*, and protein recovery *R* after foaming were most conclusive to distinguish and evaluate F-Tag performance with regard to enzyme workup. Initial activity and foamability were overall acceptable for most constructs and could be improved by manipulation of the expression system or by technical means, respectively. Enrichment *E* severely depends on the foam drainage which is dependent on the applied physical and technical setup of foam fractionation and therefore has a less general value as a performance parameter (Keshavarzi et al. 2022b). For each of the chosen three parameters, *δA*, *δm*, and *R*, we considered a threshold value of 40% appropriate to designate promising performance. Residual activity and protein recovery should be above and protein loss below this threshold. Applying those criteria to the initial set of Bla-F-Tag constructs left SlyD as the most promising F-Tag candidate. Including the linker R1, R1-HsbA, R1-Rsn-2, and R1 alone gave constructs crossing all thresholds, but Bla-R1-Rsn-2 was inferior in *δA*. Consequently, we chose Bla-SlyD, Bla-R1-HsbA, and Bla-R1 for further investigation.

### Foam fractionation of tagged Bla and eGFP

**Fig. 5:**
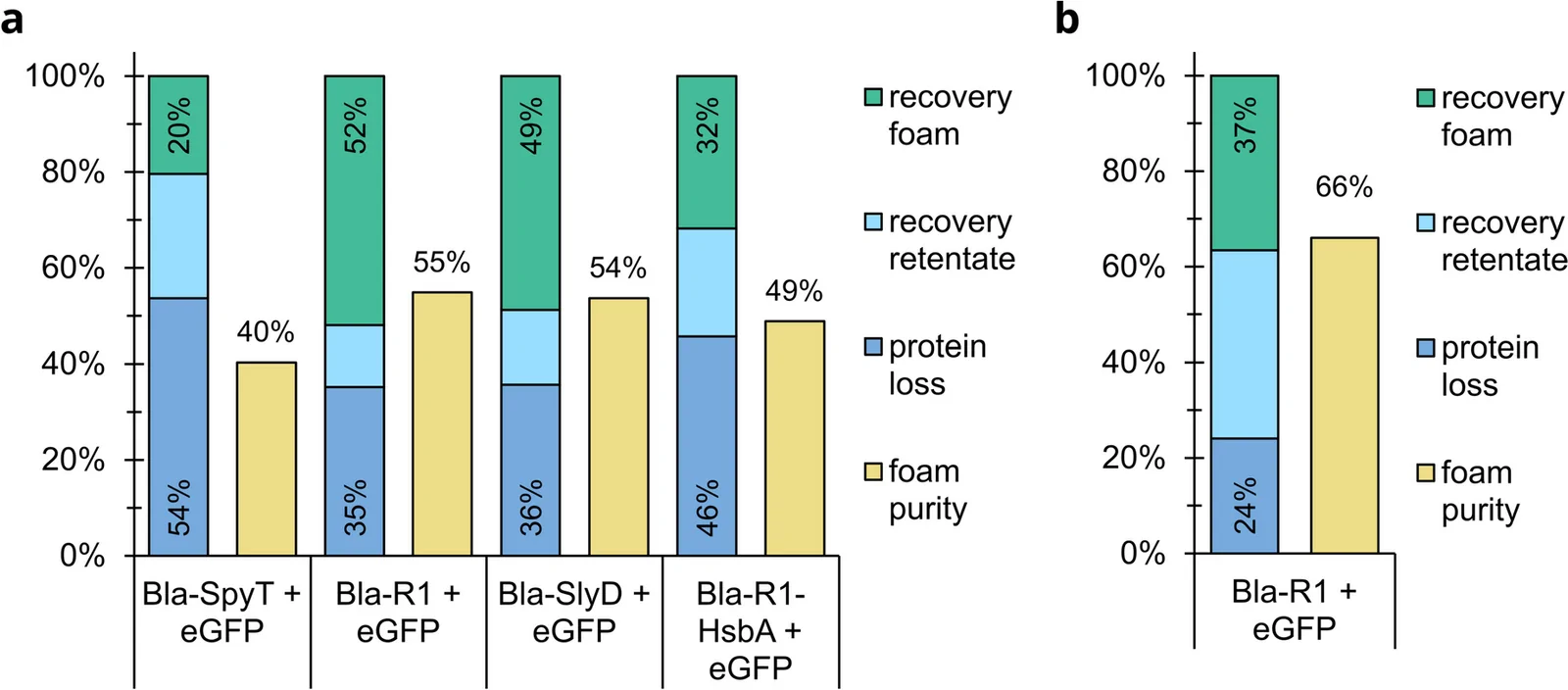
Recovery, protein loss, and foam purity after foam fractionation in a simple column with 10 mL initial volume and an air flow rate of 20 mL min^−1^; foaming proceeded until no foam overflowed. **a** Bla with different tags and eGFP as impurity; initial total protein concentration: 0.2 mg mL^−1^ at a Bla construct to eGFP ratio of 1:1; initial purity: 50% (±5%); **b** Bla-R1 and eGFP as impurity; initial total protein concentration: 0.1 mg mL^−1^ at a Bla construct to eGFP ratio of 1:1

 Bla-SlyD, Bla-R1-HsbA, and Bla-R1 were mixed with recombinant Green Fluorescent Protein (eGFP) in a mass ratio of 1:1 and subjected to foam fractionation in our simple column. However, a preferential take-up of the tagged enzyme into the foam, and thus, a purifying effect was hardly apparent (Fig. 5a). With Bla-SlyD and Bla-R1, the purity *P* in the extract was slightly above the initial value of 50%. With Bla-R1-HsbA, *P* slightly decreased below the initial value. Additionally, the recovery *R* of Bla-SlyD and Bla-R1-HsbA was significantly lower in the presence of eGFP than in its absence. At the same time, more protein than without eGFP was lost during the foam fractionation process. Bla-R1 showed a slightly better *R* after foam fractionation, but also a prominent increase in *δm*. Reduction of the initial volume and thus an increased foam height and longer drainage time had no positive effect on purity or recovery. Likewise, decreasing the initial amount of eGFP to a ratio of 33% or 25% did not improve the purity *P*_*Bla* − *R*1_. However, reducing the total initial protein concentration *c*_*initial*_ from 0.2 to 0.1 mg mL^−1^ increased *P*_*Bla* − *R*1_ to 66% (Fig. 5b).

**Fig. 6:**
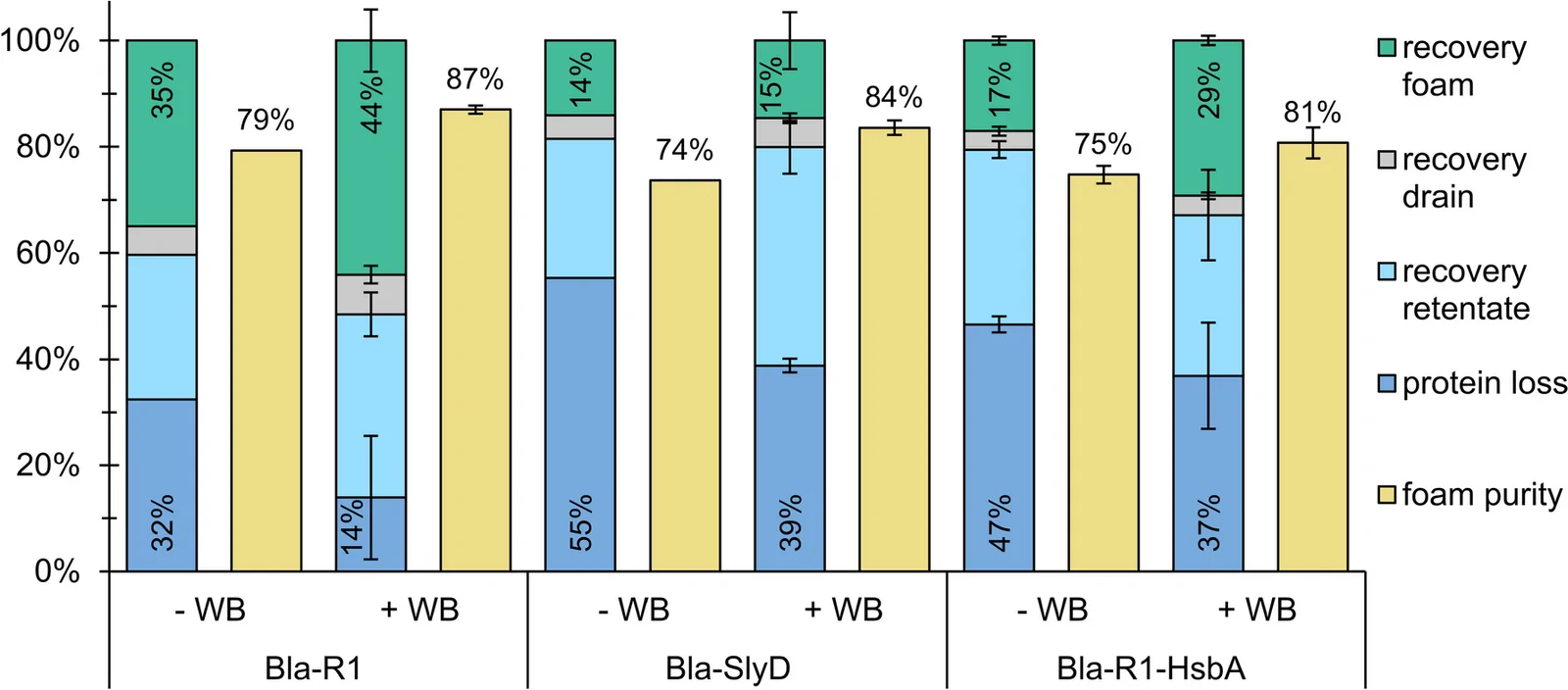
Recovery, protein loss, and foam purity after foam fractionation of Bla constructs and eGFP in an extended column with 42 mL initial volume and an air flow rate of 100 mL min^−1^ without (-WB) or with addition of wash buffer (+WB; 0.1 mol L^−1^ KPi, pH 7.5); foaming proceeded until no foam overflowed. Initial protein concentration: 0.1 mg mL^−1^at a Bla construct to eGFP ratio of 1:1; initial purity 50% (±5%). Error bars refer to two separate experiments

For all tested constructs, an overall better *P* was obtained (Fig. 6) when foam fractionation was performed in an extended column (Fig. 3b). The addition of wash buffer to the foam during foam fractionation further increased *P*. Thus, a purity up to 87% was reached without further optimization. Interestingly, with respect to all three parameters, purity, recovery, and protein loss, Bla-R1 gave the best results.

**Fig. 7:**
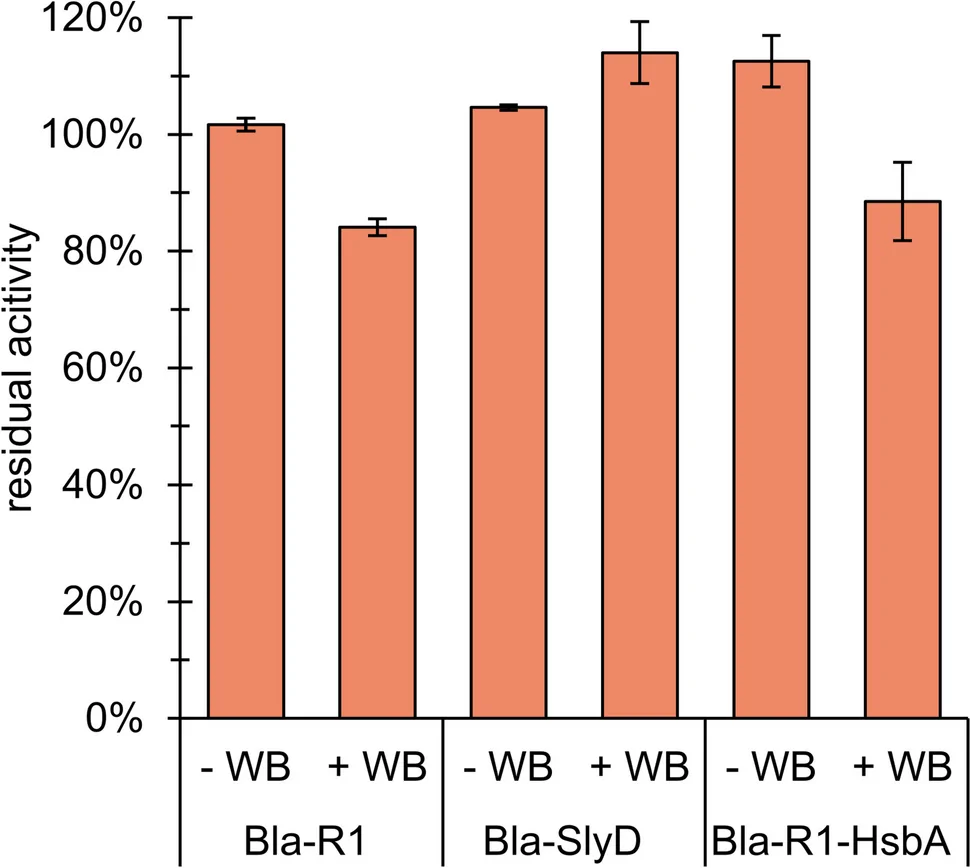
Residual activity *δA* of Bla constructs in the liquefied foam fractions after fractionation in the extended column with 42 mL initial volume and an air flow rate of 100 mL min^−1^ with and without the addition of wash buffer (WB; 0.1 mol L^−1^ KPi, pH 7.5)

In contrast, Bla-R1 performed worst regarding *δA*, and *δA* was considerably affected by the addition of wash buffer (Fig. 7). The best and even increased *δA* after the application of wash buffer displayed Bla-SlyD, while *δA* of Bla-R1-HsbA was best when the wash buffer was absent. Notably, however, *δA* of all constructs was above 80% with or without addition of wash buffer.

### F-Tag performance with PGA and RjFDH

 Fusion of SlyD, R1-HsbA, and R1, respectively, to penicillin G acylase from *Kluyvera cryocrescens* (PGA) or formate dehydrogenase from *Rhodococcus jostii* (RjFDH) gave results comparable with Bla regarding purity *P*, recovery *R*, and protein loss *δm* after foam fractionation in the simple column (Fig. S4 and S5). In both cases, constructs with R1 performed best.

Introduction of the extended column and addition of wash buffer overall increased the foam purity (Fig. S6 and S8). The effect was particularly pronounced upon addition of wash buffer. The absolute values were different for the constructs with different target proteins. For RjFDH, *P* up to 80% was obtained (Fig. S8). For PGA, *P* up to 90% was measured (Fig. S6). Notably, however, PGA constructs with R1 and SlyD could not build stable foam at a concentration of 0.05 mg mL^−1^. *δA* was again lowest for constructs with R1 after the addition of wash buffer. Nevertheless, *δA* of all constructs was above 80 % (Fig. S7 and S9).

## Discussion

There are numerous reports in the literature about proteins that are able to stabilize foam. In some cases, the ability is linked to the natural function of the proteins, e.g., stabilization of horse sweat (sLatherin (Vance et al. 2013)) or the foam nests of frogs (Rsn-2 (Fleming et al. 2009)). Predominantly, however, foam stabilization is only a secondary property, such as in the case of the best-known foam stabilizers used in food production, casein, and ovalbumin (Mohanty et al. 1988; Dickinson 1989). The only molecular property common to the various proteins appears to be surface amphiphilicity (Zayas 1997; Malmsten 1998), which is often accompanied by post-translational modifications such as glycosylation or phosphorylation (Shental-Bechor and Levy 2008). Some proteins such as Rsn-2 and BslA acquire the ability to stabilize foam only through drastic conformational changes upon contact with the air-liquid interface (Fleming et al. 2009; Bromley et al. 2015; Brandani et al. 2017). The spectrum of proteins that could in principle be used as tags for foam fractionation is therefore large. Accordingly, a broad selection of F-Tag candidates differing in functions, structures, and sizes were included in this study.

The inability to produce most of the directly fused F-Tag-Bla fusion proteins suggests a strong interaction between the two protein parts during production. Apparently, their separate preparation and only subsequent fusion reduce this interaction. The resulting constructs can be regarded as hetero-multimer-like structures with two concrete functional domains and a connecting Spy domain (Fig. 2). In addition, Bla-Rsn-2 in this constellation showed a much higher catalytic activity $\delta {\overline{A}}_{initial}$ than Bla-Rsn-2_original_, i.e., in direct fusion with Rsn-2 (Table 2; Krause et al. 2022). Together with the observation that all fusion products had a distinctly higher $\delta {\overline{A}}_{initial}$ than Bla-Rsn-2_original_, this indicated a generally beneficial effect of this construction scheme for the production of enzymes with an F-Tag.

Nevertheless, all F-Tag candidates decreased $\delta {\overline{A}}_{initial}$ to some extent. The structural models of the fusion products (Fig. 2) indicate that this could be a consequence of both direct interaction between the functional domains and shielding of the active site by the F-Tag. Also, the presence of the Spy system could naturally influence $\delta {\overline{A}}_{initial}$ as previously described for smaller fusion proteins (Panek et al. 2013; Pajęcka et al. 2014). However, since the Spy system was consistently present in our constructs, this can hardly explain the different *δA*_*initial*_ of the fusion proteins. The highest $\delta {\overline{A}}_{initial}$ occurred when the F-Tag was on the opposite side of the molecule from the active site (Rsn-2) or when there was no direct contact of the domains (ChpE, R1) (Table 2) and, at the same time, the tag size was relatively small (Table 1). Large F-Tags close to the active site but without direct interaction with the active site (BslA, HsbA) decreased $\delta {\overline{A}}_{initial}$. Constructs in which the F-Tags appear to interact punctually with the active center of Bla (NrdJ-S, R1-RdlA, SlyD) had the lowest $\delta {\overline{A}}_{initial}$. Certainly, the structural models of the fusion constructs used here must be handled with great caution with regard to the relative orientation of the domains and direct interactions of the structural units. Since structural data of the full constructs were not available, the software could only model the domains (Bla, F-Tag, SpyT/SpyC) individually and intelligently estimate orientation and interaction from these. Consequently, the PAE plots of the full constructs are also only partially good, with very good values for the separate domains and worse values for the interaction of domains (Fig. S2). However, since this approach represents relatively well the actual production of the fusion constructs using the Spy system, we assume that the models allow our cautious interpretation.

The residual activity *δA* of the fusion proteins after foaming does not appear to be related to the relative orientation of the domains or the direct interaction of the F-Tag with the active site of the Bla. Even though Bla-ChpE with the highest $\delta {\overline{A}}_{initial}$ also had the highest *δA* Bla-Rsn-2 with a comparably high $\delta {\overline{A}}_{initial}$ obtained the worst *δA* of all initial constructs (Table 2). Instead, Bla-R1-RdlA, Bla-SlyD, and Bla-R1 performed very well. Notably, all four constructs with a higher *δA* are characterized by a comparably long linker sequence connecting the globular enzyme with the F-Tag domain. Assuming that the F-Tag domains preferably binds to the air-liquid interface, this implies that the distance, at which the active enzyme is kept from the F-Tag, and thus, the interface, plays a major role for activity retention. This also implies only weak interactions between the enzyme and the aforementioned F-Tags resulting in a vastly different structure at the interface compared to the structure in solution. This fits with the hypothesized mechanism of protein denaturation at interfaces (Clarkson et al. 1999), whereby proteins undergo partial conformational changes at interfaces that turn internal hydrophobic regions outward. In the case of enzymes, this can lead to conformational changes of the active site and thus deactivation (Bechtold and Panke 2012). Consequently, preventing direct contact with the interface should reduce the loss of activity. This is supported by the observation that the introduction of the linker R1 between Bla and F-Tag generally led to an increase in *δA* (Table 2, Fig. S1). R1 could be particularly effective at maintaining a finite distance between the interfaces of a foam film because of its rigidity. A relation to the tag size is not obvious. Our assumption that the F-Tag candidates studied here have a stronger tendency to bind to the interface is justified by the observation that the fusion constructs exhibited a significantly better foamability (Fig. 4b) than the native enzyme. Bla alone is not capable of stabilizing foam in our experimental setup (Krause et al. 2022).

Under this assumption, the protein loss *δm* after foaming would also be primarily due to the individual behavior of the F-Tag domains at the interface. Certain conformational changes might favor aggregation. Unfortunately, our structural models are not suitable to describe conformations of the F-Tag at the air-water interface because they are based only on crystal structures of solubilized proteins. For BslA and Rsn-2, drastic conformational changes due to contact with the air-water interface are known in the literature (Bromley et al. 2015; Morris et al. 2016; Brandani et al. 2017), but with respect to the other F-Tag candidates we did not find any information. Interestingly, however, we observed the highest *δm* with BslA, but the lowest with Rsn-2 demonstrating that not the extent of the rearrangement process, but the tendency of the adopted conformation to aggregate probably decides *δm*. Considering this, the spacing of the proteins at the interface could also affect the magnitude of *R* as vicinity might promote aggregation. This might also mean that, at comparable concentrations, smaller molecules could outcompete larger ones because they could occupy larger distances from each other. Under our experimental conditions, however, no correlation was observed between *R* and the size of the F-Tag domains. At comparable *δm*, small and large F-Tag domains resulted in comparable *R*. In fact, *R* of Bla-SlyD and Bla-R1 was almost identical, although they were the biggest and smallest constructs, respectively, in this research.

Then again, a low *δm* not necessarily ensured a high protein recovery *R* after foaming, although *δm* above 40% ensured a low *R*. With Rsn-2, *R* was only about 22% despite the negligible *δm*. *R* describes the amount of protein found in the total liquefied foam related to the total amount of protein that initially was introduced into foaming. Apart from *δm*, this parameter depends on the tendency of the fusion construct to adsorb at the air-liquid interface. Thus, a low *R* at low *δm* indicates only a low adsorption tendency as large amounts of protein remain in the reservoir solution. Our results show that this adsorption behavior cannot be described by the influence of the fusion constructs on the surface tension, since there are hardly any differences (Fig. 4a). It must be kept in mind, of course, that in the tensiometer we used, the sorption process of large molecules is dominated by diffusive transport to the interface. In the case of rising bubbles, convective transport overrules diffusive transport, and thus, the adsorption rate on rising bubbles can be orders of magnitude faster (Keshavarzi et al. 2022a). In any case, however, our results do not reflect the assumption reported in the literature that Rsn-2 has a special N-terminal sequence that directs the protein to the air-water interface (Brandani, 2017). Such a function would have to result in Rsn-2 showing a particularly large tendency to adsorb, whereas we observed very low *R* and *δm*, i.e., large amounts of the protein remained in the retentate. It is also assumed in the literature that the tendency of proteins to adsorb at interfaces is generally greatest when they have a net charge close to their isoelectric point (pI) (Noel et al. 2002; Lambert et al. 2003). In our study, however, we did not find any correlation between the distance of the pH of the reservoir solution from the calculated pI of the respective fusion construct or F-Tag domains and *R* (Table S1). From our observations, it could be hypothesized that to obtain high *R* the F-Tag must have the ability to adsorb fast and irreversible at the gas-liquid interface while keeping the structural change to a minimum to reduce the risk of aggregation. However, it remains unclear which structural properties would be required to promote this or which physical parameters might describe the phenomenon.

Unexpectedly, the fusion constructs that exhibited the best *δA*, *δm*, and *R* after foam formation were poor performers in terms of separating Bla constructs from eGFP by foam fractionation in a simple setup. It is most likely related to poor drainage, which causes the entrainment of larger amounts of liquid, and consequently a foam purity that is little different from the purity of the reservoir solution. Probably, drainage is limited by the formation of cohesive films at the air-liquid interface (Clark et al. 1989; Clarkson et al. 1999), which results from the presumed interactions of F-Tag domains. These are most likely further enhanced by interactions with eGFP. The latter can be inferred from the observation that a mixture of eGFP and Bla-SpyT, neither of which alone exhibited significant interactions with the interface or the ability to stabilize foam, produced a stable foam in the separation experiments (Fig. 5a). Drainage limitation also resulted from the increased total protein concentration in the purification batch compared to the previous foaming experiments, which causes the formation of thicker films at the interface that hinder drainage (Zayas 1997; Boonyasuwat et al. 2003; Keshavarzi et al. 2022b). Indeed, we observed that the purity *P* of the Bla fusion constructs significantly improved after the total protein concentration was halved (Fig. 5b). At the same time, higher stability of the foams generated by the constructs (Fig. 4b) correlated with lower *P*. This correlation between foam stability and *P* was also evident for the fusion constructs with PGA and RjFDH (Fig. S4 and S5). The fusion constructs of both alternative target enzymes basically formed less stable foams than the comparable constructs with Bla, but at the same time exhibited a high *P*. Nevertheless, the eGFP content remained high, i.e., drainage was insufficient here as well.

As a logical consequence, higher purity *P* was achieved in all approaches by creating the possibility to add wash buffer to the foam, which replaced the liquid entrained in the foam (Keshavarzi et al. 2022a). The modified geometry of the extended column also had a positive effect on *P*. It has long been known that column geometry and process parameters significantly affect the results of foam fractionation (Wong et al. 2001; Koop et al. 2021; Oraby et al. 2022). In our case, most likely the longer and atypical geometry of the overflow path resulted in improved drainage and thus reduced volume of entrained liquid. At the same time, however, *δm* increased probably due to the longer foaming time and thus longer contact with the interface. During the rise of the foam, the liquid content is reduced due to drainage which leads to thinning of the plateau channels between the gas bubbles and thus closer contact of the molecules at the interface with neighboring molecules and interfaces. The elongated foaming time can finally lead to more extensive unfolding, aggregation, and precipitation.

Fortunately, this effect was counteracted by the addition of wash buffer, by filling the drained channels with fluid and stabilization of the distance between the lamella. This resulted in shorter and less extensive contact between molecules and interfaces. With the wash buffer, only a small portion of the fusion constructs were removed into the drain, indicating a stronger adsorption to the gas-liquid interface than eGFP. Thus, we achieved high *P* at overall low *δm* with all three selected F-Tag candidates and all three model enzymes.

In conclusion, our results show that a surprisingly wide range of proteins that have the natural ability to stabilize foam enable activity-preserving recovery of Bla after foaming by serving as a fusion tag. The enormous structural differences between these proteins indicate that the actual performance in foam fractionation is due less to punctate than to global motifs or to constellations of motifs. Thus, for the residual activity of the target enzyme after foam formation, the distance between target enzyme and fusion tag seems to play a predominant role. Protein loss appears to be primarily related to the conformation of the fusion tag adopted at the interface, while relatedly, the extent of recovery of fusion constructs from the foam is significantly influenced by the tendency to adsorb and interact at the interface. On the separation of contaminating proteins, the generation of lower foam stabilities has a positive effect, and the use of wash buffers is mandatory for a good separation result.

Surprisingly, the most promising results in all respects were obtained with the R1 sequence, which was originally introduced only as a linker structure between the target enzyme and various F-Tag candidates. Its small size and simple helical structure offer good opportunities to better understand and specifically optimize the behavior of this tag in foam fractionation through observations at the interface and local structural changes. At the same time, it presents little difficulty in expression in microbial hosts and shows little effect on the catalytic activity of the target enzymes studied to date. Thus, there may also be the potential to extend this sequence into a foam fractionation tool that operates independently of individual molecular properties of the target enzymes. Further studies are needed to clarify the influence of more complex protein mixtures on foam fractionation performance. Moreover, the influence of other components of the fermentation media, especially sugars and salts, to which the purification process will ultimately be applied, will have to be investigated. At the moment, we continue to believe that the use of F-Tags offers good prospects for the development of an efficient, surfactant-free technology for enzyme purification.

## Supplementary information

ESM 1 (PDF 2317 kb)

## Data Availability

Data will be made available upon reasonable request to the corresponding author.
