# Supplementary material for: Foam fractionation Tags (F-Tags) enabling surfactant-free, activity-preserving recovery of enzymes
Source: Appl Microbiol Biotechnol. 2024 Jan 17;108:140. doi: 10.1007/s00253-023-12837-1 (PMC10794386; doi:10.1007/s00253-023-12837-1)
Supplement: Supplementary file 1 [file 253_2023_12837_MOESM1_ESM.pdf]

Applied Microbiology and Biotechnology

Supplementary information

Foam fractionation Tags (F-Tags) enabling surfactant free, activity-preserving recovery of enzymes

Thomas Krause[1], Behnam Keshavarzi[2], Sascha Heitkam[2], Marion B. Ansorge-Schumacher[1*]

[1]Department of Molecular Biotechnology, TU Dresden, Dresden, Germany

[2]Institute of Process Engineering and Environmental Technology, TU Dresden, Dresden, Germany

Corresponding author: Prof. M. B. Ansorge-Schumacher, e-mail: marion.ansorge@tu-dresden.de, telephone: +49 351 463 39519, ORCID ID: 0000-0002-2912-546X

Supplementary Information

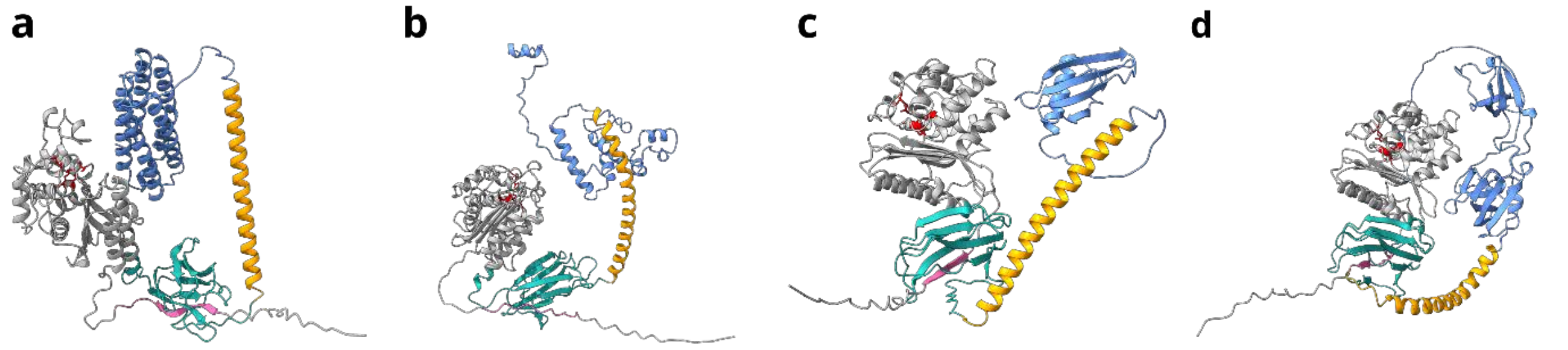

Fig. S1: Structural models of fusion constructs between Bla and F-Tags. green: SpyCatcher-domain; pink: SpyTag-domain; gray: Bla with catalytic residues (Ser70, Lys73, Ser130; Glu166 (Minasov et al. 2002)) in red; orange: R1-Linker; blue: F-Tag: **a)** Bla-R1-HsbA **b)** Bla-R1-NrdJ-S **c)** Bla-R1-Rsn-2 **d)** Bla-R1-SlyD.

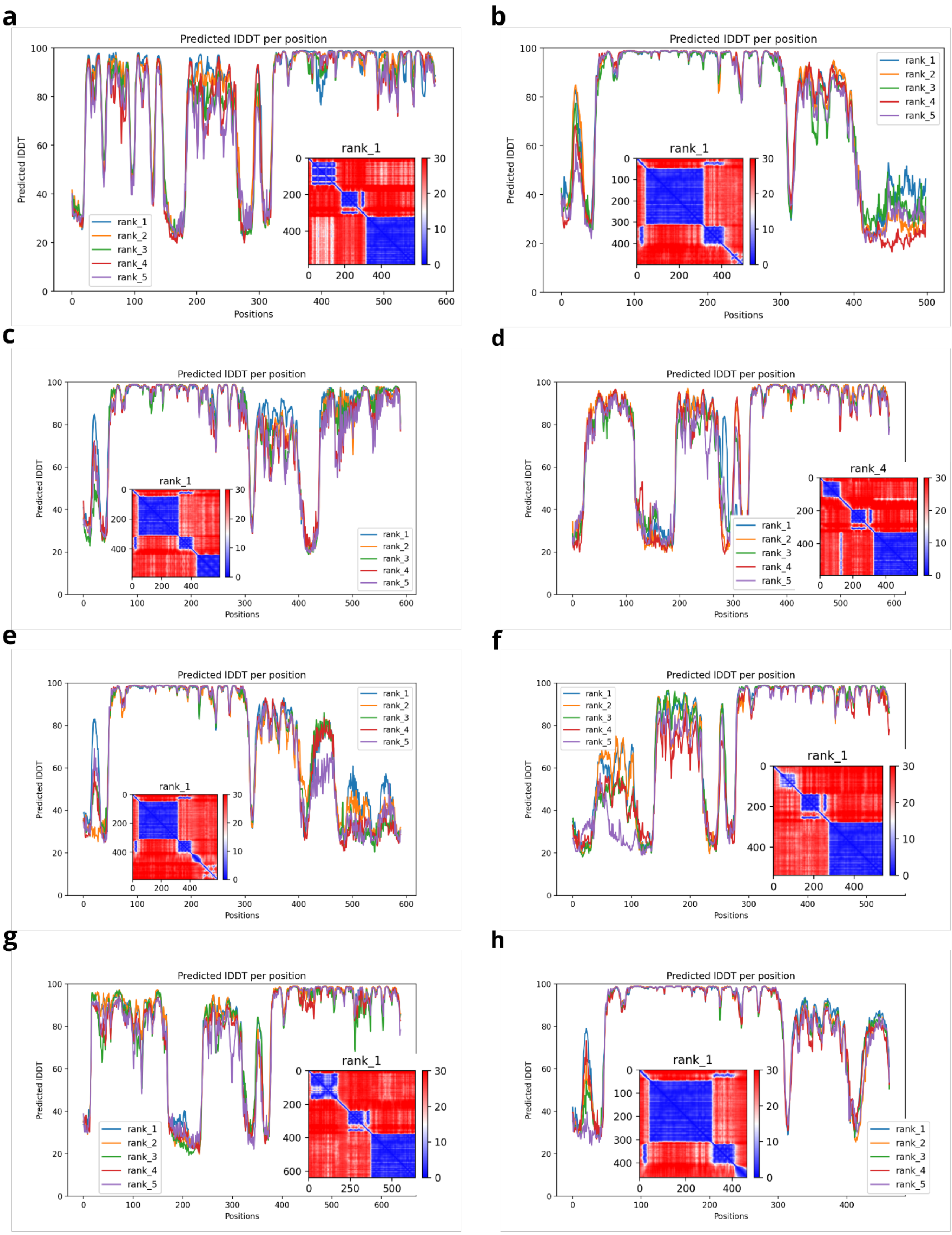

Fig. S2: pLDDT and PAE plot for structures predicted by AlphaFold2 using the ColabFold interface for: **a)** Bla-BslA **b)** Bla-ChpE **c)** Bla-HsbA **d)** Bla-NrdJ-S **e)** Bla-R1-RdlA **f)** Bla-Rsn-2 **g)** Bla-SlyD **h)** Bla-R1

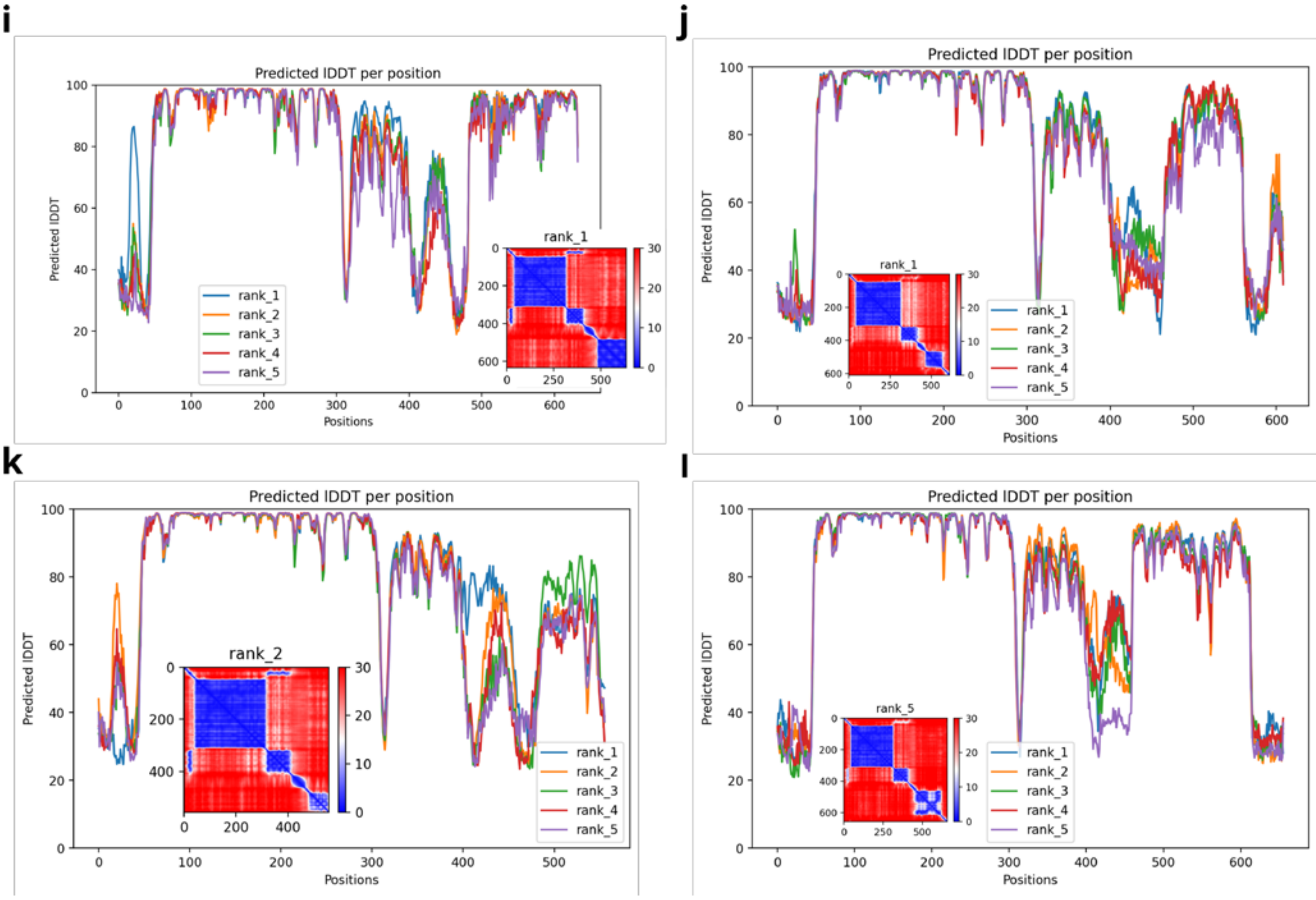

Fig. S3: pLDDT and PAE plot for structures predicted by AlphaFold2 using the ColabFold interface for: **i)** Bla-R1-HsbA **j)** Bla-R1-NrdJ-S **k)** Bla-R1-Rsn-2 **l)** Bla-R1-SlyD.

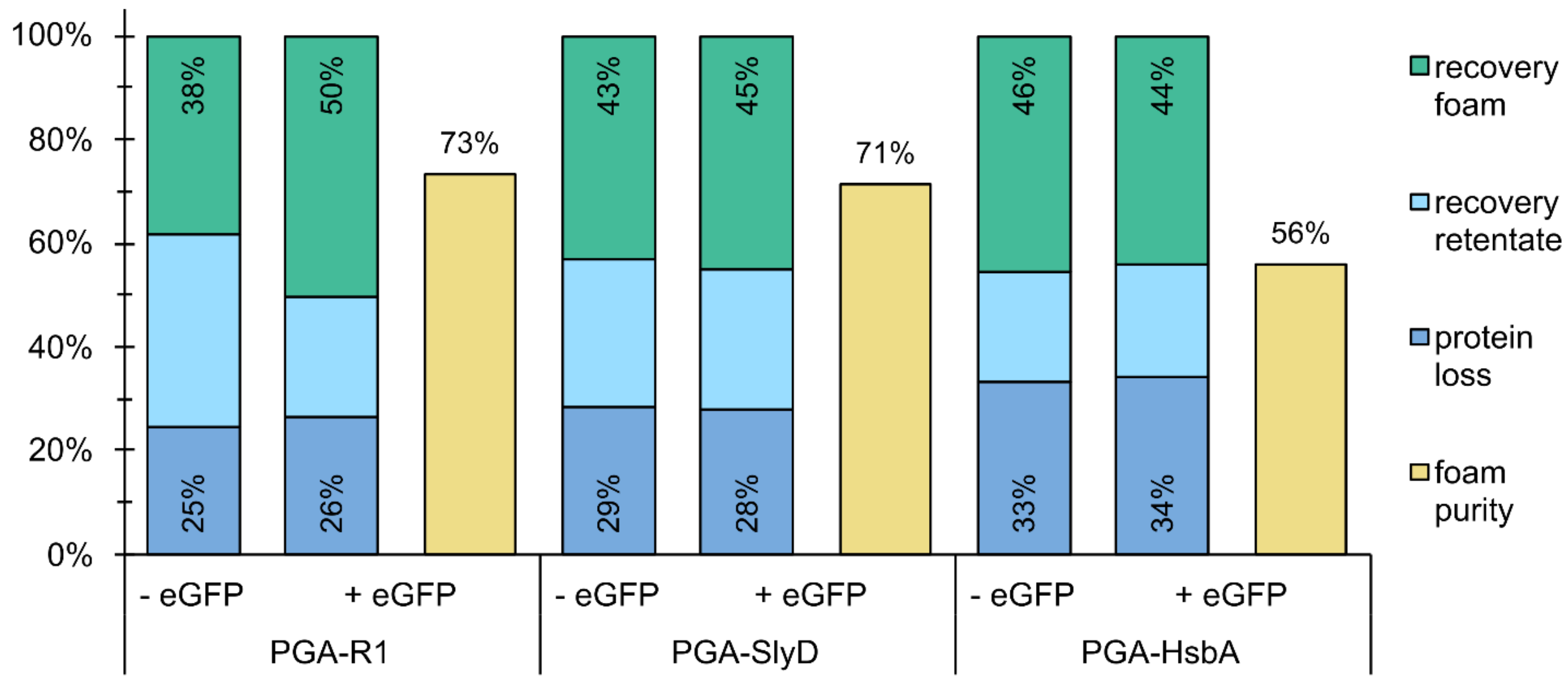

Fig. S4: Recovery, protein loss and foam purity after foam fractionation of PGA fusion constructs and eGFP as impurity in a simple column with 10 mL initial volume and an air flow rate of 20 mL·min^{-1}; foaming proceeded until no foam overflowed. Initial total protein concentration: 0.2 mg·mL^{-1} at a PGA construct to eGFP ratio of 1:1; initial purity: 50% (±5%).

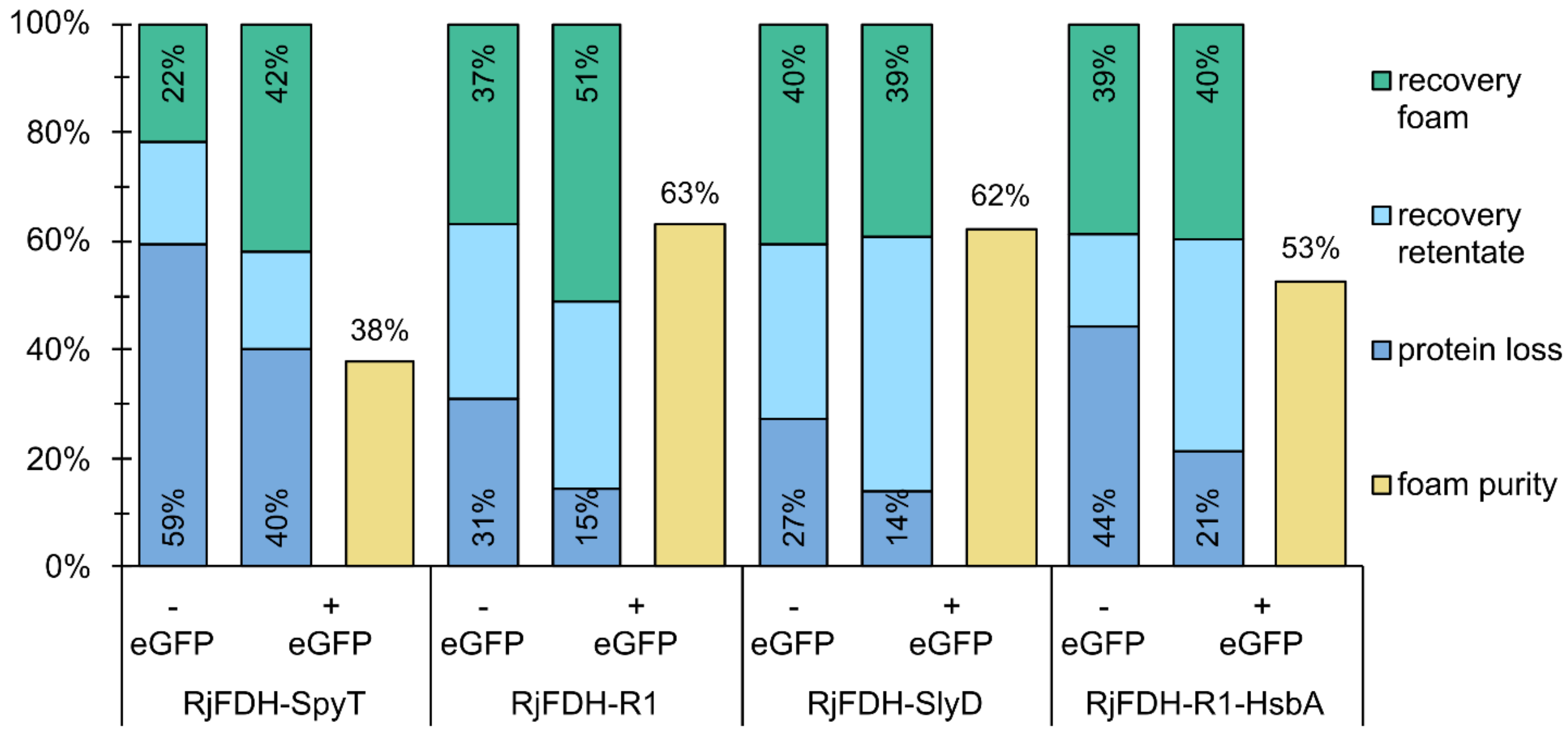

Fig. S5: Recovery, protein loss and foam purity after foam fractionation of RjFDH fusion constructs and eGFP as impurity in a simple column with 10 mL initial volume and an air flow rate of 20 mL·min^{-1}; foaming proceeded until no foam overflowed. Initial total protein concentration: 0.2 mg·mL^{-1} at an RjFDH construct to eGFP ratio of 1:1; initial purity: 50% (±5%).

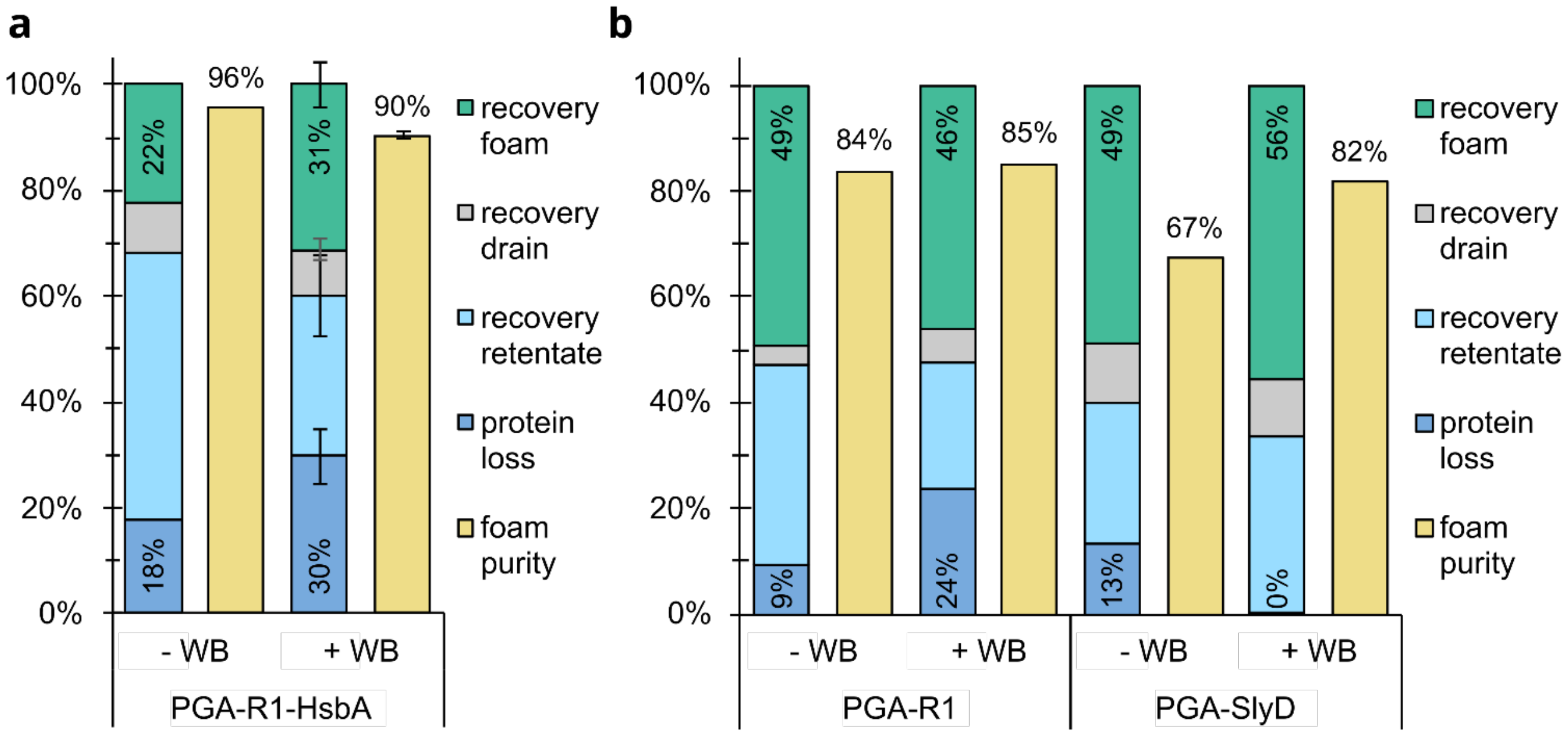

Fig. S6: Recovery, protein loss and foam purity after foam fractionation of PGA fusion constructs and eGFP in an extended column with 42 mL initial volume and an air flow rate of 100 mL·min^{-1} with or without addition of wash buffer (WB; 0.1 mol·L^{-1} KPi, pH 7.5); foaming proceeded until no foam overflowed. **a)** Initial protein concentration: 0.1 mg·mL^{-1} at a PGA construct to eGFP ratio of 1:1; **b)** Initial protein concentration: 0.2 mg·mL^{-1} at a PGA construct to eGFP ratio of 1:1; initial purity 50% (±5%).

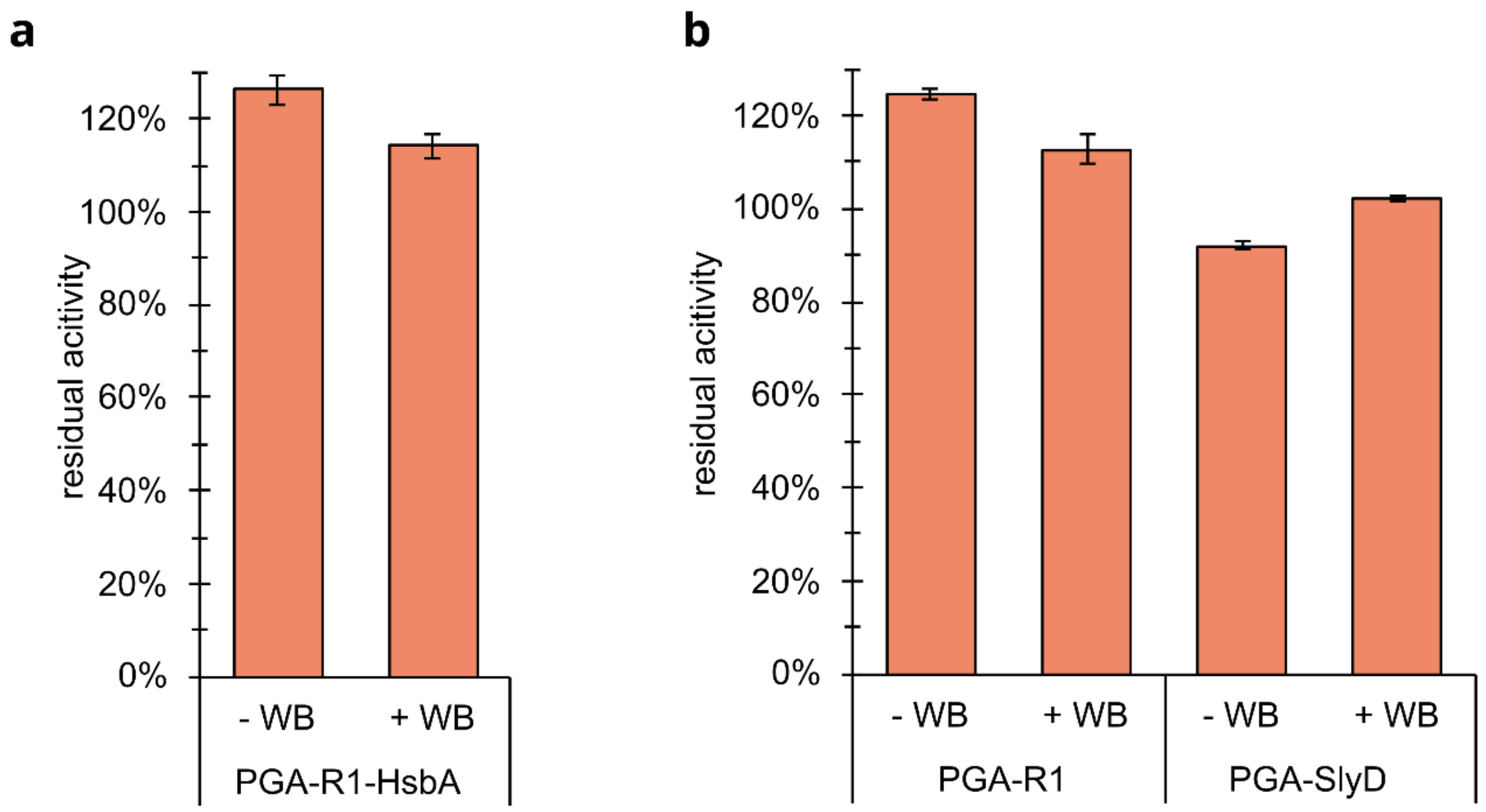

Fig. S7: Residual activity δA of Bla fusion constructs in the liquefied foam fractions after fractionation in the extended column with 42 mL initial volume and an air flow rate of 100 mL·min^{-1} with and without the addition of wash buffer (WB; 0.1 mol·L^{-1} KPi, pH 7.5). **a)** Initial protein concentration in the foam fractionation: 0.1 mg·mL^{-1} **b)** Initial protein concentration in the foam fractionation: 0.2 mg·mL^{-1}.

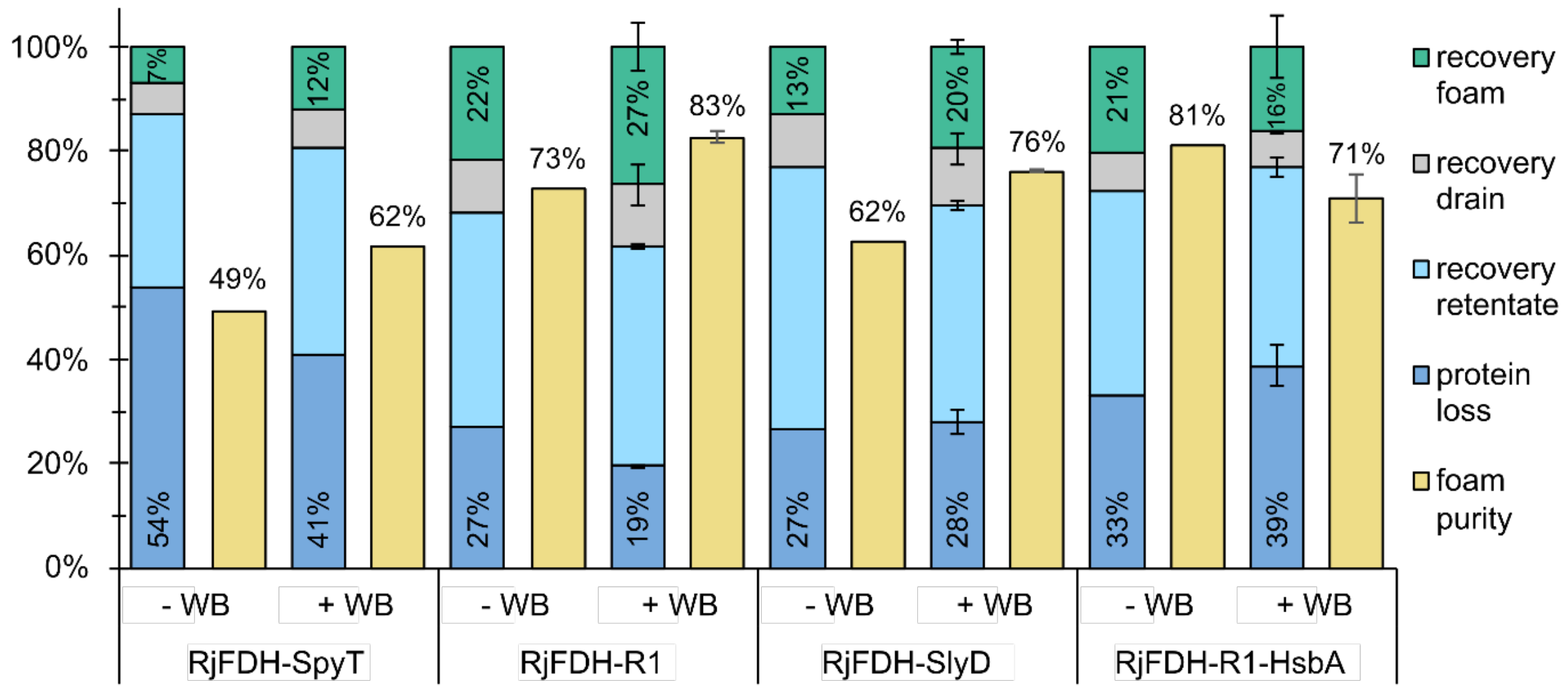

Fig. S8: Recovery, protein loss and foam purity after foam fractionation of RjFDH fusion constructs and eGFP in an extended column with 42 mL initial volume and an air flow rate of 100 mL·min^{-1} with or without addition of wash buffer (WB; 0.1 mol·L^{-1} KPi, pH 7.5); foaming proceeded until no foam overflowed. Initial protein concentration: 0.1 mg·mL^{-1}at a RjFDH construct to eGFP ratio of 1:1; initial purity 50% (±5%).

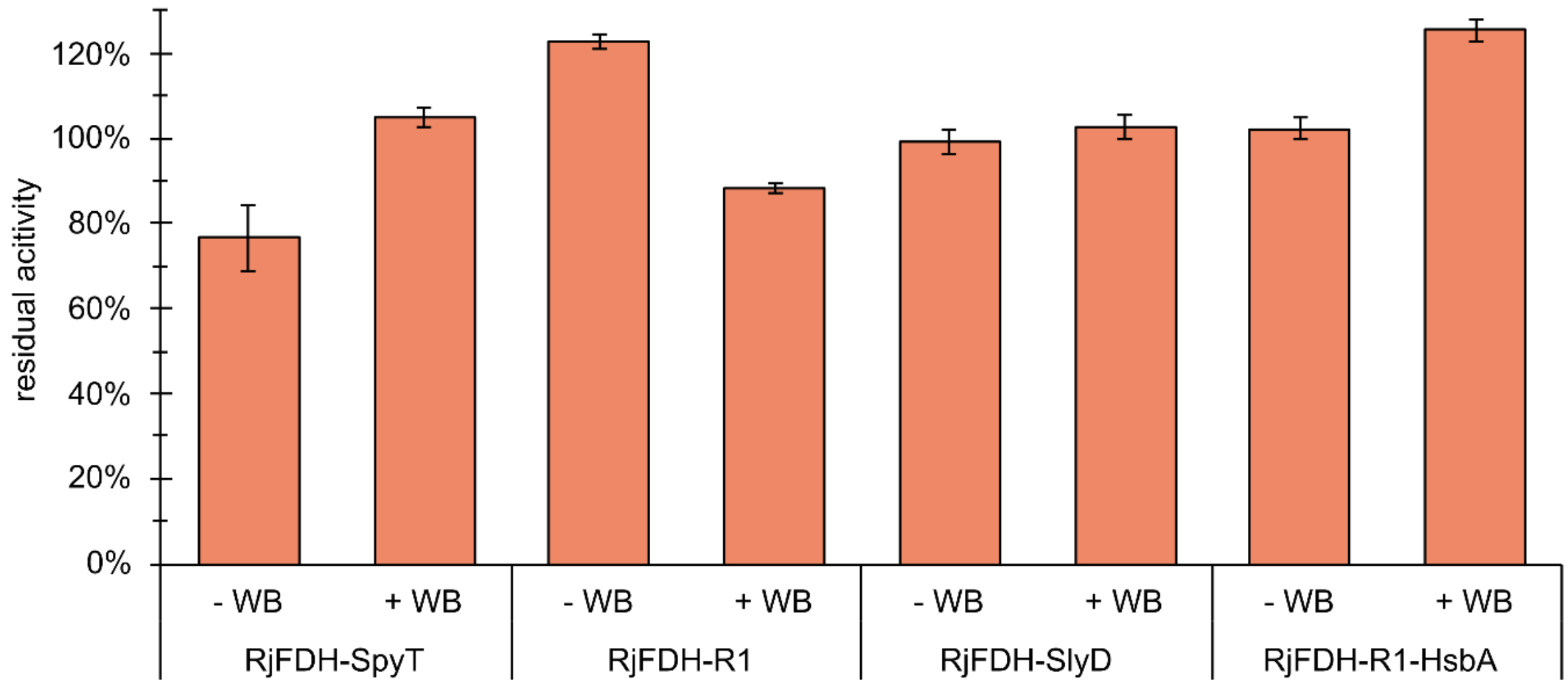

Fig. S9: Residual activity δA of RjFDH fusion constructs in the liquefied foam fractions after fractionation in the extended column with 42 mL initial volume and an air flow rate of 100 mL·min^{-1} with and without the addition of wash buffer (WB; 0.1 mol·L^{-1} KPi, pH 7.5).

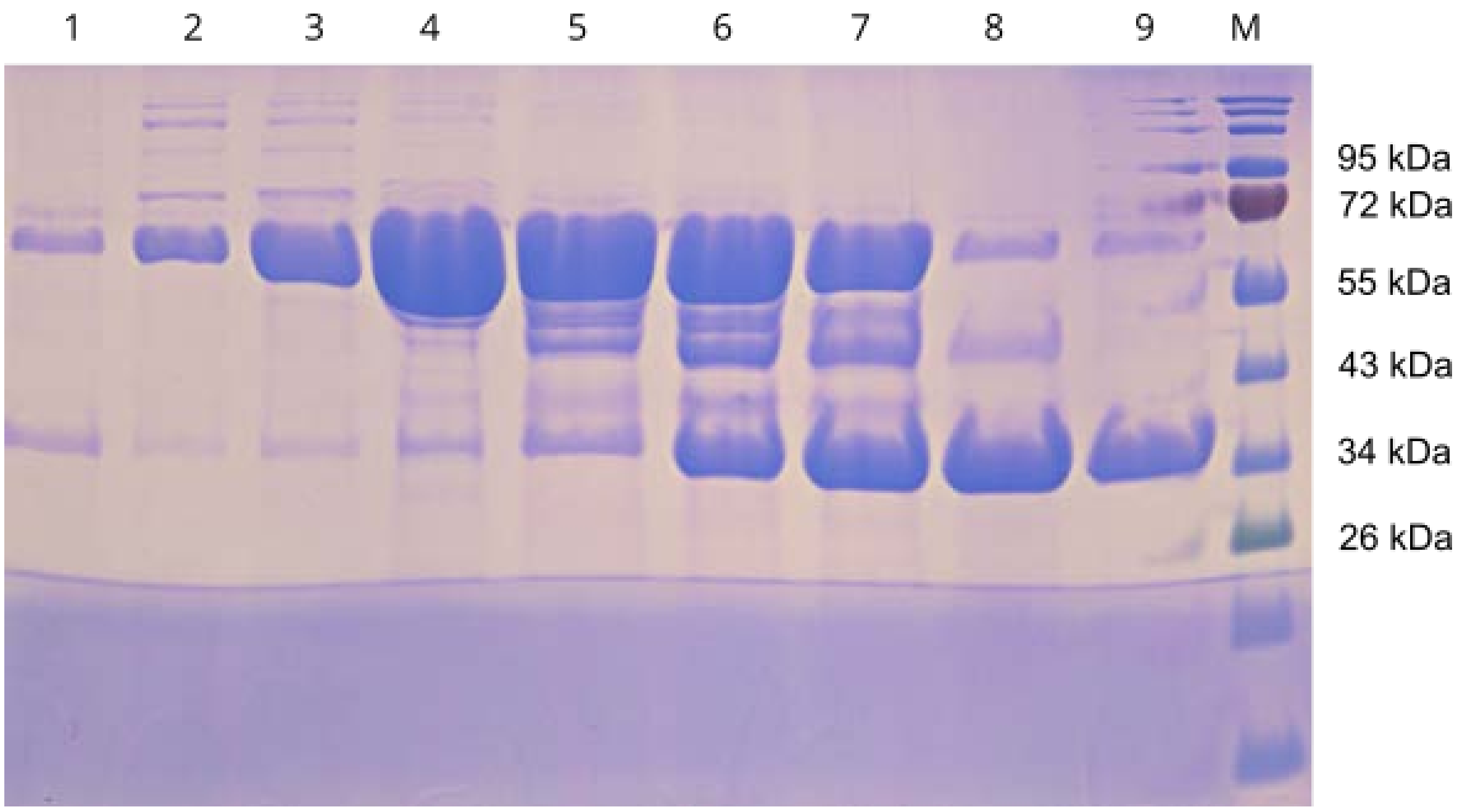

Fig. S10: SDS-PAGE to observe to purification success after size exclusion chromatography of Bla-R1-Rsn-2; 1-9: Elution fractions, M: Color Prestained Protein Standard, Broad Range (New England Biolabs, USA); band at 60 kDa corresponds to Bla-R1-Rsn-2, band at 34 kDa corresponds to unreacted Bla-SpyT.

Table S1: Calculated isoelectric points (pI), ΔpH and recovery R for F-Tags and Bla-F-Tag constructs. pIs were calculated using the Expasy ProtParam tool (https://web.expasy.org/protparam/). ΔpH is calculated as $\Delta pH = |7.5 - pI|$.

Fusion protein	pI (F-Tags)	ΔpH (F-Tags)	pI (Fusion proteins)	ΔpH (Fusion proteins)	R [%]
**Bla-BslA**	9.59	2.09	6.15	1.35	15
**Bla-ChpE**	9.70	2.20	6.23	1.27	16
**Bla-HsbA**	4.55	2.95	5.68	1.82	52
**Bla-NrdJ-S**	4.77	2.73	5.26	2.24	32
**Bla-R1-RdlA**	4.60	2.90	5.50	2.00	37
**Bla-Rsn-2**	6.28	1.22	5.61	1.89	22
**Bla-SlyD**	5.10	2.40	5.29	2.21	64
**Bla-R1-HsbA**	8.26	0.76	6.12	1.38	64
**Bla-R1-NrdJ-S**	4.77	2.73	5.61	1.89	52
**Bla-R1-Rsn-2**	4.47	3.03	5.44	2.06	34
**Bla-R1-SlyD**	5.23	2.27	5.84	1.66	61
**Bla-R1**	4.93	2.57	5.42	2.08	21
